# Transmission of MRSA, ESBL *E. coli*, and *C. difficile* within a tertiary care hospital and across surrounding facilities in Japan: a molecular epidemiological study with the PCR-based Open-reading frame typing

**DOI:** 10.1017/ice.2024.178

**Published:** 2025-01

**Authors:** Hiroki Saito, Satoshi Miike, Tatsuya Ohno, Momoko Anzai, Fumimasa Kasai, Akiko Hosoyama, Tomomi Takakura, Yosuke Tanaka, Shigeki Fujitani

**Affiliations:** 1 Department of Emergency and Critical Care Medicine, St. Marianna University School of Medicine, Kawasaki, Kanagawa, Japan; 2 Interdepartmental Division of Critical Care Medicine, University of Toronto, Toronto, Ontario, Canada; 3 Department of Emergency and Critical Care Medicine, St. Marianna University Yokohama Seibu Hospital, Yokohama, Kanagawa, Japan; 4 Department of Clinical Laboratory, St. Marianna University Yokohama Seibu Hospital, Yokohama, Kanagawa, Japan; 5 Department of Nursing, St. Marianna University Yokohama Seibu Hospital, Yokohama, Kanagawa, Japan

## Abstract

**Objective:**

To determine the regional impact of transmission of multidrug-resistant organisms (MRDOs) and *Clostridioides difficile* (*C. difficile*) among a tertiary care hospital and surrounding facilities including long-term care facilities (LTCFs).

**Design:**

Retrospective cohort study.

**Methods:**

Patients admitted to a tertiary care hospital from July 2019 to July 2021 were recruited if their clinically collected cultures grew the following pathogens: Methicillin-resistant *Staphylococcus aureus* (MRSA), Extended-Spectrum Beta-Lactamase (ESBL) producing Enterobacterales, *Pseudomonas aeruginosa* with difficult-to-treat resistance, Carbapenem-resistant Enterobaterales, Vancomycin-resistant Enterococci, and *C. difficile*. Patient characteristics including admission and discharge pathway were collected. For the isolates of MRSA, ESBL-producing *Escherichia coli* (*E. coli*), and *C. difficile*, a molecular epidemiological analysis was conducted, utilizing the PCR-based Open-Reading Frame Typing (POT) method.

**Results:**

Three hundred-five patients were identified with a total of 332 culture specimens of the target pathogens. The top three were 132 MRSA isolates (43.3%, out of 305), 97 ESBL *E. coli* (31.8%), and 32 ESBL Enterobacterales (non-*E. coli*) (10.5%). The target pathogens were more detectable within 3 days among patients admitted from LTCFs or other hospitals than those admitted from home (Odds Ratio 4.6, 95% confidence interval 2.8-7.6, p-value < 0.001). The molecular epidemiological analysis suggested the transmissions of MRSA, ESBL *E. coli* and *C. difficile* occurred 52 out of 111 patients within the in-hospital environment, and 7 out of 128 within the prehospital environment, respectively.

**Conclusions:**

MDROs/*C. difficile* transmission is prevalent within a tertiary care hospital and further complicated by its inter-facility transmission across surrounding LTCFs and hospitals in Japan.

## Introduction

The global burden of bacterial antimicrobial resistance (AMR) is estimated to be 1.27 million deaths each year.^1^ The Government of Japan adopted an AMR national action plan in 2016 to respond to this public health threat more comprehensively. In light of the aging society, one of the six key strategies in this action plan is focused on AMR related research, including the one within long-term care facilities (LTCFs).^2,3^ LTCF residents are known to disproportionately harbor multidrug-resistant organisms (MDROs).^4–6^ In Japan, about 40% of LTCF residents is reported to carry multidrug-resistant Gram-negative bacteria in their oral cavities.^7^ Furthermore, facility-level infection control of LTCFs is not systemically assessed in Japan.^8^ While LTCFs may be a potential reservoir of MDROs, the epidemiological interaction and impact of MDROs between LTCFs and acute care hospitals is yet to be clarified in Japan.

The polymerase chain reaction (PCR)-based Open-reading frame (ORF) Typing (POT) method, originally developed in Japan, is a novel method to detect DNA polymorphisms.^9–12^ While epidemiological interaction of MRDOs is typically assessed by pulse pulsed-field gel electrophoresis (PFGE), multi-locus sequencing typing (MLST), or whole genome sequencing (WGS), they are time-consuming and resource-intensive procedures.^13^ The POT method exhibits strain identification capabilities using multiplex PCR.

In this study, we applied the POT method to investigate the regional epidemiological interaction of MRDOs between a tertiary care hospital and surrounding LTCFs and hospitals, including intra-facility and inter-facility transmission.

## Methods

### Study design and setting

This was a retrospective cohort study of patients admitted to a 518-bed tertiary care hospital in Yokohama, Japan, with a mean length of hospital stay of 10.7 days. Four infectious disease specialists, including the authors (HS and MS) were on staff full-time. At the hospital, Methicillin-resistant *Staphylococcus aureus* (MRSA) of all isolated *Staphylococcus aureus*, Extended-Spectrum Beta-Lactamase (ESBL)-producing *Escherichia coli* (*E. coli*) of all isolated *E. coli* accounted for 44.6% and 22.0%, respectively. Implementation of hand hygiene in the facility was consistent with World Health Organization (WHO) guidelines^14^. Contact precaution was implemented for all patients known to be colonized/infected with target MDROs and *Clostridioides difficile* (*C. difficile*). Routine active surveillance of MDROs on admission was not included in the hospital policy. Decolonization of MRSA was not performed routinely. During the study period, despite the challenges of the COVID-19 pandemic, the hospital’s infection prevention control measures overall remained unchanged, and there were no MDRO outbreaks requiring active investigation. Hand hygiene compliance improved from 50% in 2019 to >80% in 2020 likely due to the COVID-19 pandemic, and remained > 70% in 2021 (appendix).

### Study participants

Patients were eligible for the study if the following criteria were met: 1. patients were admitted to the hospital between July 1st, 2019 and July 31^st^, 2021; 2. microbiological cultures collected clinically during hospitalization grew the target pathogens, that is, MRSA, ESBL-producing *E. coli*, ESBL Enterobacterales (non-*E. coli*), *Pseudomonas aeruginosa* with difficult-to-treat resistance (PA-DTR),^15^ Carbapenem-resistant Enterobacterales (CRE),^16^ Vancomycin-resistant Enterococci (VRE), and *C. difficile*. Patient characteristics were collected by medical chart review including age, sex, medical procedures, patient location before admission, detailed ward movement during hospitalization, length of hospital stay, and discharge status.

### Microbiological testing

The microbiological testing was conducted by the certified microbiologists (TO, MA and YT) in accordance with national standards (appendix).

### POT method

The POT method is a method of genetic characterization by detecting approximately 20 ORFs using multiplex PCR.^9^ These target ORFs were determined by conducting a comparative analysis of the entire genomic sequences of multiple strains of the target bacterial species. POT codes were converted from the results of electrophoretic band patterns obtained by substituting the presence or absence of the respective target ORFs, described as a combination of numerical values like POT1-POT2(-POT3). In this study, the following POT kits were utilized for MRSA, ESBL *E. coli* and *C. difficile*, respectively: Cica Geneus Staph POT kit, Cica Geneus ESBL Genotype Detection kit, and Cica Geneus Toxin Gene Detection kit (Kanto Chemical Co., Ltd., Tokyo, Japan).

### Epidemiological interaction

In the analysis of epidemiological interaction, patients hospitalized for less than 3 days before culture collection vs for 3 days or more were considered to have the potential for prehospital vs for in-hospital contact, respectively. Prehospital contact was defined if the patient pair belonged to the same household or facility and shared the identical POT strain. In-hospital contact was defined as follows:^17^ (1) direct ward contact: the patient pair shared the identical POT strain, and was hospitalized to the same ward, overlapping for at least one calendar day within the risk period; (2) indirect ward contact: the patient pair shared the identical POT strain, and was hospitalized to the same ward, but the period of the source patient admission on the ward preceded that of another patient without an overlap within the risk period; (3) direct in-hospital contact: the patient pair shared the identical POT strain, and was hospitalized to the hospital within the same risk period, although not on the same ward; (4) no in-hospital contact.

### Status of infection prevention and control

Surrounding LTCFs and hospitals as admission source and/or discharge locations of the patients were approached by email and/or postal mail, and if agreed, visited by the trained infection control team of the tertiary care hospital to assess their status of infection control, using WHO Hand Hygiene Self-Assessment Framework (HHSAF),^18^ and Infection Prevention and Control Assessment Framework (IPCAF)^19^ as validated tools (appendix). HHSAF and IPCAF consists of 27 items (maximum score 500) and 81 items (maximum score 800), respectively, and the scores are classified into four categories according to each quartile:1. Inadequate, 2. Basic, 3. Intermediate, and 4. Advanced.^14,20^

### Statistics

Descriptive analysis reporting frequencies and proportions were used for patient characteristics including prehospital or in-hospital contact. Univariate analysis was conducted to assess the association between categorical variables, calculating odds ratios (ORs) with 95% confidence intervals (CIs). Pearson χ2 test and Mann Whitney test were applied to compare categorical variables and continuous variables among two groups, respectively. All statistical analyses were performed using SPSS Version 27.0 (SPSS Inc., Chicago, USA) with a statistical significance of *P*-value < 0.05.

### Ethics and reporting

The study was approved by the institutional ethical review board of St. Marianna University (4411). The consent was obtained in an opt-out method.

## Results

### Study population

**Figure 1.**
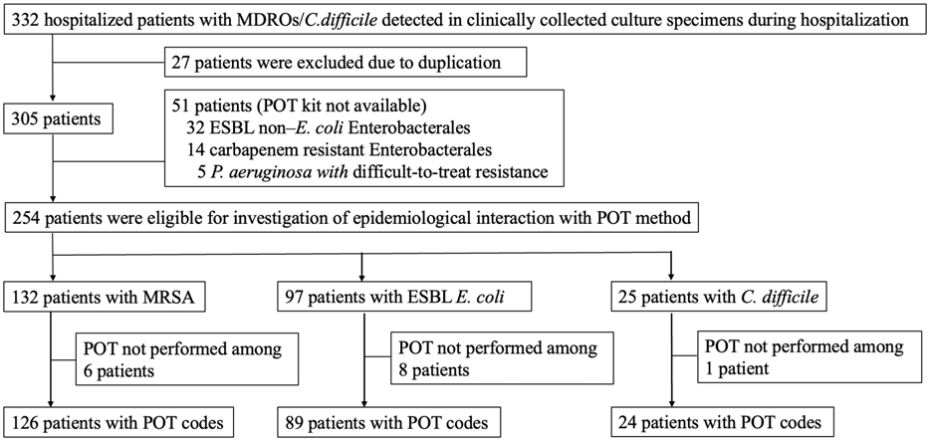
Patients included in the study. MDRO, multidrug-resistant organism; ESBL, Extended-Spectrum Beta-Lactamase; *E. coli, Escherichia coli; P. aerugionosa, Pseudomonas aeruginosa*; C. difficile, *Clostridioides difficile*; MRSA, Methicillin-resistant *Staphylococcus aureus*; POT, Polymerase chain reaction-based Open-reading frame Typing.

Three hundred-five individuals participated in the study with a total of 332 clinically obtained culture specimens of the target pathogens (Figure 1). Patient characteristics are presented in Table 1. The median age of the patients was 78 years old and the median hospital length of stay was 26 days. Of the target MDROs, MRSA was the most frequently detected (132/305, 43.3%), followed by ESBL *E. coli* (97/305, 31.8%), ESBL Enterobacterales (non-*E. coli*) (32/305, 10.5%), CRE (14/305, 4.6%), and PA-DTR (5/305, 1.6%). *C. difficile* was detected among 25 patients (8.2%). VRE was not detected. The route of admission among the 305 patients was as follows: 186 (61.0%) from home, 93 (30.5%) from 50 LTCFs, and 26 (8.5%) from 20 other hospitals. The target pathogens were significantly more likely to be detected within 2 days among patients admitted from LTCFs or other hospitals than those admitted from home (OR 4.6, 95% CI 2.8–7.6, *P*-value < 0.001). Approximately half of the patients (150/305, 49.2%) were discharged to LTCFs or transferred to other hospitals, including more than one-third of patients admitted from home (68/186, 36.6%).

**Table 1.** Demographics of patients with MDROs/*C. difficile* detected in clinically collected culture specimens

		Route of admission		
Variables	Total (N = 305)	Home (N = 186)	LTCFs (N = 93)	Hospitals (N = 26)
Age, median y (IQR)	78 (71–85)	76 (70–83)	82 (79–88)	71 (62–79)
Sex, male, N (%)	180 (59.0)	114 (61.3)	46 (49.5)	20 (76.9)
Detection of MDROs/*C. difficile*				
MRSA, N (%)	132 (43.3)	78 (41.9)	41 (44.1)	13 (50.0)
Days from admission to culture collection (IQR)	1 (1–15)	4 (1–22)	1 (0–2)	1 (0–9)
Culture specimen, N (%)				
Sputum	99 (75.0)	54 (69.2)	35 (85.4)	10 (76.9)
Blood	9 (6.8)	8 (10.3)	1 (2.4)	0 (0)
Urine	9 (6.8)	6 (7.7)	2 (4.9)	1 (7.7)
Skin	8 (6.1)	4 (5.1)	3 (7.3)	1 (7.7)
Stool	6 (4.5)	6 (7.7)	0 (0)	0 (0)
Bile	1 (0.8)	0 (0)	0 (0)	1 (7.7)
ESBL *E. coli*, N (%)	97 (31.8)	53 (28.5)	38 (40.9)	6 (23.1)
Days from admission to culture collection (IQR)	1 (0–7)	2 (1–19)	0.5 (0–1)	1.5 (0–4)
Culture specimen, N (%)				
Urine	58 (59.8)	30 (56.6)	24 (63.2)	4 (66.7)
Stool	12 (12.8)	8 (15.1)	2 (5.3)	2 (33.3)
Blood	11 (11.3)	5 (9.4)	6 (15.8)	0 (0)
Sputum	11 (11.3)	5 (9.4)	6 (15.8)	0 (0)
Skin	2 (2.1)	2 (3.8)	0 (0)	0 (0)
Bile	2 (2.1)	2 (3.8)	0 (0)	0 (0)
Ascites	1 (1.0)	1 (1.9)	0 (0)	0 (0)
*C. difficile*, N (%)	25 (8.2)	22 (8.6)	2 (2.2)	1 (3.8)
Days from admission to culture collection (IQR)	15 (9–27)	13 (9–26)	79	25
Culture specimen, N (%)				
Stool	25 (100)	22 (100)	2 (100)	1 (100)
ESBL Enterobacterales (non-*E. coli*), N (%)	32 (10.5)	15 (8.1)	11 (11.9)	6 (23.1)
Days from admission to culture collection (IQR)	3 (1–34)	31 (3–43)	1 (1–4)	1 (1–13)
CRE, N (%)	14 (4.6)	14 (7.5)	0 (0)	0 (0)
Days from admission to culture collection (IQR)	7 (4–11)	7 (4–11)		
PA-DTR, N (%)	5 (1.6)	4 (2.2)	1 (1.1)	0 (0)
Days from admission to culture collection (IQR)	124 (1–130)	128 (94–150)		
VRE, N (%)	0 (0)			
Outcome				
Deaths, N (%)	68 (22.3)	33 (17.7)	25 (26.9)	10 (38.5)
Length of hospital stay, median d (IQR)	26 (12–51)	35 (18–61)	14 (6–26)	27 (11–46)
Discharge destination, N (%)				
Home	87 (28.5)	85 (45.7)	0 (0)	2 (7.7)
LTCFs	43 (14.1)	3 (1.6)	38 (40.9)	2 (7.7)
Hospitals	107 (35.1)	65 (34.9)	30 (32.3)	12 (46.2)

Note. LTCF, long-term care facility; IQR, interquartile range; MRSA, Methicillin-resistant *Staphylococcus aureus*; ESBL, Extended-Spectrum Beta-Lactamase; *E. coli*, *Escherichia coli*; *C. difficile*, *Clostridioides difficile*; CRE, Carbapenem-resistant Enterobacterale; PA-DTR, *Pseudomonas aeruginosa* with difficult-to-treat resistance; VRE, Vancomycin-resistant Enterococci.

### Molecular epidemiological analysis with POT method

**Figure 2.**
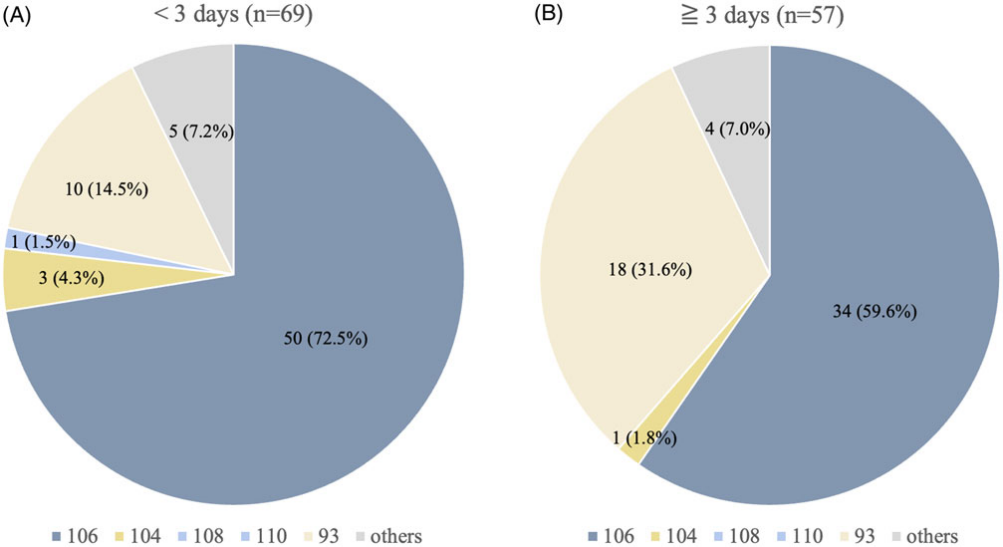
POT1 value of MRSA according to the timing of culture collection. CA-MRSA, community-acquired Methicillin-resistant *Staphylococcus aureus*; HA-MRSA, hospital-acquired Methicillin-resistant *Staphylococcus aureus*. (A) Cultures collected within 2 days of admission. (B) Cultures collected after 3 days of admission.

POT tests were performed for 239 specimens among a total of 254 patients with MRSA, ESBL *E. coli*, and *C. difficile*, after excluding 15 patients whose specimens were not processed for logistic and technical reasons (ie, poor bacterial growth from the original specimens). The POT codes, composed of POT1-POT2-POT3, revealed 58 distinct MRSA strains among 126 patients, 70 in ESBL *E. coli* among 89 patients, and 20 in *C. difficile* among 24 patients, respectively. The number of patients who shared the same POT codes with any of the other patients was 82 (65.1%) in MRSA, 29 (32.6%) in ESBL *E. coli*, and 7 (29.2%) in *C. difficile*, respectively. The POT codes of the 126 MRSA are shown in Figure 2. MRSA with POT1 values of 104/106/108/110, representing *SCCmec* type IV, accounted for 70.6%, and POT1 93, representing *SCCmec* type II, for 22.2%. Patients whose MRSA cultures were collected within 2 days of hospitalization had a greater association with the detection of MRSA with POT1 104/106/108/110 (OR 2.3, 95% CI 1.0–4.9, *P*-value 0.039), whereas patients whose cultures collected after 3 days of hospitalization had a greater association with the detection of MRSA with POT1 93 (OR 2.7, 95% CI 1.1–6.5, *P*-value 0.022).

### Epidemiological interaction of MRSA, ESBL E. coli and C. difficile

**Figure 3.**
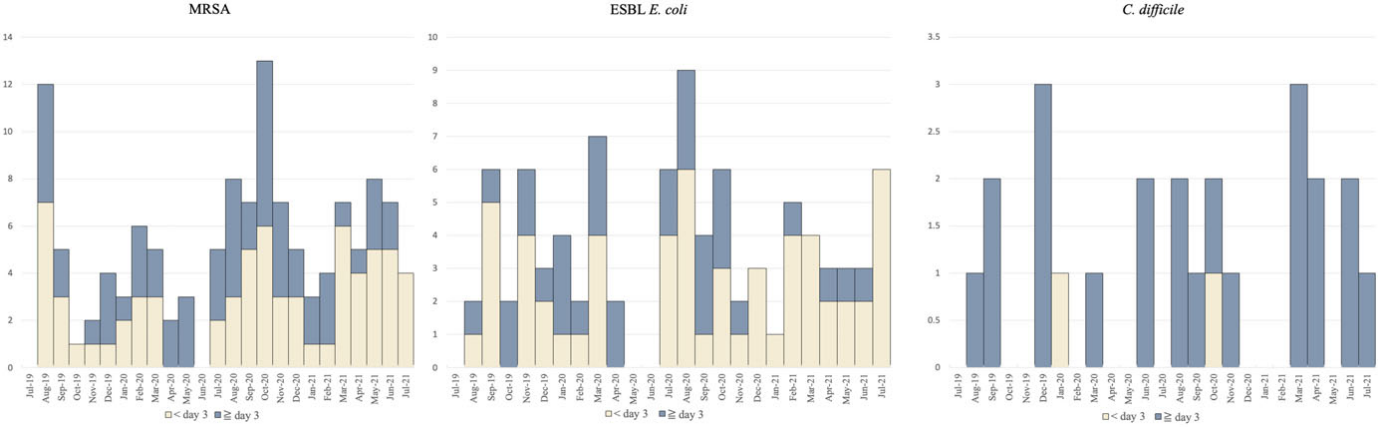
Epidemic curve of MRSA/ESBL *E. coli*/*C. difficile* according to the timing of culture collection. MRSA, Methicillin-resistant Staphylococcus aureus; ESBL *E. coli*, Extended-Spectrum Beta-Lactamase producing *Escherichia coli; C. difficile, Clostridioides difficile*.

Epidemic curves based on the number of days from admission to culture collection (< 3 d or ≧ 3 d) are shown in Figure 3. The number of patients with prehospital contact and in-hospital contact by each MRSA/ESBL *E. coli*/*C. difficile* is shown in Table 2.

**Table 2.** Epidemiological interaction of MRSA/ESBL *E. coli*/*C. difficile* via the POT method

	MRSA (N = 126)	ESBL *E. coli* (N = 89)	*C. difficile* (N = 24)
Prehospital contact			
Culture collection < 3 days of hospitalization, N	69	57	2
Patients who shared POT codes, N	44	16	0
Admitted from the same LTCFs, N (%)	7 (15.9)	0 (0)	n/a
Admitted from the same hospitals, N (%)	0 (0)	0 (0)	n/a
No prehospital contact, N (%)	37 (84.1)	16 (100)	n/a
In-hospital contact			
Culture collection ≧ 3 d of hospitalization, N	57	32	22
Patients who shared POT codes, N	35	11	6
Direct ward contact^a^, N (%)	12 (34.3)	0 (0)	1 (16.7)
Indirect ward contact^b^, N (%)	3 (8.6)	1 (9.1)	1 (16.7)
Direct hospital contact^c^, N (%)	4 (11.4)	3 (27.3)	0 (0)
No in-hospital contact, N (%)	16 (45.7)	7 (63.6)	4 (66.7)

Note. MRSA, Methicillin-resistant *Staphylococcus aureus*; ESBL E. coli, Extended-Spectrum Beta-Lactamase producing *Escherichia coli*; *C. difficile*, *Clostridioides difficile*; POT; Polymerase chain reaction-based Open-reading frame Typing; LTCF, long-term care facility.

a If the patient pair shared the identical POT strain of MDRO, was admitted to the same ward, overlapping for at least one calendar day within the risk period.

b If the patient pair shared the identical POT strain of MDRO, was admitted to the same ward, but the period the source patient admission in the ward preceded that of the acquisition patient without overlap within the risk period.

c If the patient pair shared the identical POT strain of MDRO, was admitted to our hospital within the risk period, although not the same ward.

One hundred twenty-eight patients out of 239 (53.6%) with MRSA/ESBL *E. coli*/*C. difficile* had the target pathogens detected within 2 days of admission to the hospital. As for MRSA, 44 out of 69 patients shared the same POT codes. Of these, 11 patients had been in the same LTCFs prior to admission, among whom 7 shared the same POT codes within the same LTCFs, suggesting prehospital contact. The POT codes 106-183-37 strain was identified in 5 out of the 7 prehospital contact. As for ESBL *E. coli*, among 16 patients who shared the same POT codes, no one shared the prehospital contact.

Among 111 patients whose cultures were collected after 3 days of hospital admission, 52 patients shared POT types. As for MRSA, in-hospital contact (ie, direct ward, indirect ward or direct hospital contact) was indicated in one-third of patients (19/57, 33.3%), including 11 of the 43 patients (25.6%) admitted from home. Among the 19 patients with in-hospital contact and the remaining 16 patients with no recorded in-hospital contact, 15 and 9 were linked to POT1 codes 104/106/108/110 while 3 and 7 were linked to POT1 code 93, respectively. The POT codes 106-183-37 strain was identified in 11 out of 19 with in-hospital contact.

There were 13 patients with direct ward contact sharing the same POT codes (12 MRSA, and 1 *C. difficile*, respectively) (Table 3). The 13 patients with direct ward contact had a longer median length of hospital stay compared to 98 patients without direct ward contact (34.0 vs 14.5 days, *P*-value < 0.005). The 13 patients with direct ward contact received the following care during hospitalization: indwelling urinary catheter (9 patients), tracheal intubation or tracheostomy (3), and central venous catheter (3).

**Table 3.** Characteristics of patients with direct ward contact

									Direct			
No	Age	Sex	Culture specimen	Detected pathogen	POT code	Ward	Intubation/ Tracheotomy	Urinary catheter	Ward contact (days)	Ward contact with	Room contact (days)	Room contact with
1	80s	M	Sputum	MRSA	93-137-35	ICU	Yes	Yes	9	Patients 2, 3	No	
2	70s	F	Sputum	MRSA	93-137-35	ICU	Yes	Yes	9	Patients 1, 3	No	
3	80s	M	Sputum	MRSA	93-137-35	ICU	No	Yes	9	Patients 1, 2	No	
4	70s	M	Sputum	MRSA	106-183-37	HCU	Yes	Yes	25		No	
5	70s	M	Sputum	MRSA	106-183-37	general	No	Yes	13		No	
6	80s	M	Sputum	MRSA	106-183-37	HCU	No	No	8	Patient 4	No	
7	70s	F	Stool	MRSA	106-183-37	general	No	No	5	Patient 4	No	
8	70s	M	Sputum	MRSA	106-183-37	general	No	Yes	29	Patient 7	No	
9	40s	M	Sputum	MRSA	106-183-37	HCU	No	Yes	17		15	
10	80s	M	Sputum	MRSA	106-183-37	general	No	No	12	Patient 8	12	Patient 8
11	80s	F	Sputum	MRSA	106-183-53	general	No	Yes	6		No	
12	70s	M	Blood	MRSA	106-129-1	HCU	No	Yes	4		No	
13	70s	M	Stool	*C. difficile*	4-347	general	No	No	3		No	

Note. M, male; F, female; POT, Polymerase chain reaction-based Open-reading frame Typing; MRSA, Methicillin-resistant *Staphylococcus aureus*; *C. difficile*, *Clostridioides difficile*; ICU, intensive care unit; HCU, high care unit.

### Status of infection prevention and control at surrounding facilities

HHSAF and IPCAF were assessed for two LTCFs and two hospitals between December 2021 and October 2022. In the two LTCFs, the HHSAF scores were 125 (Inadequate) and 222.5 (Basic) while the IPCAF scores were 265 (Basic) and 310 (Basic), respectively. In the two hospitals, the HHSAF scores were 280 (Intermediate) and 305 (Intermediate) while the IPCAF scores were 440 (Intermediate) and 770 (Advanced), respectively.

## Discussion

In this study, the regional impact of intra-facility and inter-facility transmission of MRDOs/*C. difficile* among a tertiary care hospital and surrounding facilities was assessed in Japan. The unique healthcare setting in Japan was described in the patient population: the majority of the patients were the elderly; about 40% of the patients were admitted from surrounding LTCFs or hospitals; they were hospitalized for as long as a month; and more than a third of those originally admitted from home were not discharged back home. The molecular epidemiological analysis with POT method revealed that more than half of the target pathogens were detected early after admission, suggesting that MDROs/*C. difficile* were carried into the tertiary hospital from prehospital environments. In addition, the analysis confirmed that in-hospital contact (ie, intra-facility transmission) was a common pathway of transmission in the context of a relatively longer hospital length of stay at the tertiary care hospital. About two thirds of those discharged alive were discharged or transferred to LTCFs or other hospitals where the facility-level infection control was variable. The findings reinforce the knowledge of MDROs sharing network between LTCFs and hospitals.^21,22^

Using the POT method, we evaluated the possibility of MRSA/ESBL *E. coli*/*C. difficile* transmission in prehospital contact such as the residential setting at LTCFs before admission and its transmission during hospitalization at the tertiary care hospital. The POT method presents several strengths, including the ability to yield results within approximately half a day, whereas PFGE requires several days. It also facilitates standardized procedures using commercially available kits, and the numerical representation of POT types minimizes inter-examiner variability. However, a notable limitation is that the method is confined to detecting specific predetermined ORFs, which may diminish its capacity to differentiate novel strains not anticipated during its development. In phylogenetic analysis, the discriminatory ability of the POT method may be inferior to single nucleotide polymorphism (SNP) analysis using WGS^23^ but equivalent to PFGE and MLST.^10^ Another study showed Simpson’s diversity index for *E. coli* was 0.968 in the POT method and 0.979 in PFGE.^11^

The POT method has predominantly been applied to nosocomial outbreaks of MDROs such as MRSA^24^ and *Acinetobacter baumannii*.^25,26^ Others examined the concordance of *C. difficile* strains detected within a hospital^27^ and explored strains of MRSA carried by companion animals.^28^ This study is unique in that the POT method was utilized to evaluate MDRO intra-facility transmission within a hospital during non-outbreak periods and to investigate the sharing of MDROs in the prehospital environment. Approximately one-third of in-hospital contact for MRSA were traced by the POT method, suggesting a potential burden of transmission within the tertiary care hospital. Conversely, the limited detection of prehospital MDROs sharing was partly attributed to the extensive number of facilities from which the patients were admitted (50 LTCFs and 20 hospitals).

POT1 for *Staphylococcus aureus* is composed of seven ORFs of which five are associated with *SCCmec* and two with the sequence type (ST). The combination of *SCCmec* and ST-type-dependent ORFs allows for the differentiation between conventional community-acquired MRSA (CA-MRSA) and hospital-acquired MRSA (HA-MRSA). Strains with *SCCmec* type IV, which predominantly represent CA strains, have a POT1 value of 104/106/108/110, while the New York/Japan clone (ST5, *SCCmec* type II), known for HA strains, exhibits a POT1 value of 93.^10,12^ Our research findings indicate that CA-MRSA strains were more frequently detected in specimens collected within 2 days of admission, while HA-MRSA strains were more prevalent in specimens collected after 3 days of admission. This trend is consistent with the conventional distinction between CA and HA strains. On the other hand, reports from Japan suggest that since 2010, there has been an increasing prevalence of *SCCmec* ^29^ and POT strains^30^ conventionally regarded as CA-MRSA detected in healthcare settings. Our findings of those with prehospital contact from the same LTCFs sharing the same POT codes, most commonly a CA-MRSA strain (POT 106-183-37), and those with in-hospital contact sharing the same POT codes, more than half of which was the CA-MRSA strain, suggest the epidemiological interaction may be more complex. In addition, the proportion of HA-MRSA (POT1 93) detected shortly after admission was 14.5% out of the total MRSA strains. This implies a potential burden of HA-MRSA in the prehospital environment, encompassing surrounding LTCFs and hospitals.

Among LTCFs where the assessment of HHSAF and IPCAF was conducted, the scores were generally low, Inadequate to Basic. We assessed two hospitals, one primary care and the other secondary care, and both yielded Intermediate to Advanced. A previous study across 57 hospitals in Japan reported that primary care hospitals tended to score lower in IPCAF compared to secondary or tertiary care hospitals, likely reflecting resource differences guided by the national regulatory system with financial incentives.^8^ LTCF residents are frequently hospitalized, and even after discharge, they repeatedly get readmitted.^31^ Since LTCFs and primary care hospitals often serve as a source of referrals to tertiary care hospitals and as discharge destinations, improvement in facility-level infection control at these facilities may have a large impact on the regional AMR epidemiology.

This study has several limitations. First, we included only clinically collected cultures, and did not examine potential MDRO carriers who did not develop clinical symptoms. There is no standard policy for MDRO screening in healthcare settings in Japan, and the tertiary hospital of the study doesn’t perform routine MRDO screening. Active screening may facilitate a more comprehensive evaluation of MDRO transmission. Second, we didn’t collect microbiological specimens at LTCFs and other hospitals than the tertiary care, mainly due to resource restrictions and logistic feasibility. The molecular epidemiological analysis with the POT method among residents/patients at surrounding facilities may help us better understand the inter-facility MDRO transmission. Third, due to the retrospective nature of our study, prehospital and/or postdischarge healthcare data was not systemically obtained other than locations. Fourth, because this study was conducted during the COVID-19 pandemic when the visitation to facilities was restricted due to the health policy in Japan, the number of surrounding facilities that completed the HHSAF and IPCAF assessment was limited, precluding us from making more comprehensive assessment of the association between the epidemiological analysis and the infection control measures in a region, and even at the visited facilities, the situation of infection control might not reflect that in normal times. Fifth, this study was conducted at a single institution in Japan, which may limit generalizability to other regions and/or countries.

In conclusion, MDROs/*C. difficile* transmission is possibly prevalent within an acute care hospital and across its surrounding LTCFs and hospitals in Japan. The molecular epidemiology analysis with the POT method suggested intra-facility transmission may contribute to the regional AMR epidemiology in Japan where the care dependency is high in the aging society and the hospital length of stay is long at an acute care hospital. Further research is warranted on mechanisms of MDRO transmission at LTCFs, and how to prevent and control the inter-facility transmission with a region-wide approach.

## Supporting information

Saito et al. supplementary material Saito et al. supplementary material

## Supplementary material

The supplementary material for this article can be found at https://doi.org/10.1017/ice.2024.178.
