# Supplementary material for: Transmission of MRSA, ESBL E. coli, and C. difficile within a tertiary care hospital and across surrounding facilities in Japan: a molecular epidemiological study with the PCR-based Open-reading frame typing
Source: Infect Control Hosp Epidemiol. 2024 Nov 11;46(1):35–42. doi: 10.1017/ice.2024.178 (PMC11717480; doi:10.1017/ice.2024.178)
Supplement: Saito et al. supplementary material [file S0899823X24001788sup001.docx]

**Hand hygiene compliance rate**

St. Marianna University Yokohama Seibu Hospital hand hygiene compliance rate annual summary (2018–2022).

|  | 2018 | | | 2019 | | | 2020 | | | 2021 | | | 2022 | | |
| --- | --- | --- | --- | --- | --- | --- | --- | --- | --- | --- | --- | --- | --- | --- | --- |
|  | O | A | C  (%) | O | A | C  (%) | O | A | C  (%) | O | A | C  (%) | O | A | C  (%) |
| Before touching  a patient | 269 | 138 | 51.3 | 405 | 194 | 47.9 | 1671 | 1328 | 79.5 | 1723 | 1193 | 69.2 | 1318 | 834 | 63.3 |
| Before clean/aseptic procedure | 65 | 24 | 36.9 | 78 | 35 | 44.9 | 279 | 203 | 72.8 | 247 | 139 | 56.3 | 181 | 99 | 54.7 |
| After body fluid exposure risk | 58 | 31 | 53.4 | 75 | 44 | 58.7 | 455 | 390 | 85.7 | 447 | 343 | 76.7 | 309 | 230 | 74.4 |
| After touching a patient | 271 | 149 | 55.0 | 375 | 216 | 57.6 | 1955 | 1654 | 84.6 | 2349 | 1817 | 77.4 | 1456 | 969 | 66.6 |
| After touching patient surroundings | 420 | 228 | 54.3 | 493 | 233 | 47.3 | 2196 | 1831 | 83.4 | 1705 | 1242 | 72.8 | 1214 | 842 | 69.4 |
| Summary | 1083 | 570 | 52.6 | 1426 | 722 | 50.6 | 6556 | 5406 | 82.5 | 6471 | 4734 | 73.2 | 4478 | 2974 | 66.4 |

Note. O, number of opportunities; A, number of actions; C, compliance rate.

**Microbiological testing**

Bacterial identification and drug susceptibility tests: MicroScan WalkAway-40 plus (Beckman Coulter, Inc., CA, USA)

Blood culture incubations: BACTEC FX (Becton Dickinson Co, NJ, USA)

Confirmation of MDROs: MicroScan susceptibility results in conjunction with a selective and differentiating agar, CHROMagar MRSAII (Becton Dickinson Co, Franklin Lakes, NJ, USA) for MRSA, VRE Selective Agar (Becton Dickinson Co, Franklin Lakes, NJ, USA) for VRE, a drug-containing disc, MASTDISCS Combi AmpC & ESBL (Mast Group, Ltd., Liverpool, UK) for ESBL.

Confirmation of *C. difficile*: TECHLAB C. DIFF QUIK CHEK complete kit (Kohjin Bio Co, Ltd., Saitama, Japan) and a selective and differentiating agar, CCMA agar EX (Shimazu Co, Ltd., Kyoto, Japan)

**Definitions of multi-drug resistant pathogens**

*Pseudomonas aeruginosa* with difficult-to-treat resistance (PA-DTR) was defined as *Pseudomonas aeruginosa* intermediate or resistant to all reported agents in carbapenem, β-lactam, and fluoroquinolone categories.^1^ Carbapenem-resistant Enterobacterales (CRE) was defined as members of the Enterobacterales order resistant to at least one carbapenem antibiotic or producing a carbapenemase enzyme, consistent with the definition of the United States Centers for Disease Control and Prevention (US-CDC).^2^

Reference

1. Kadri SS, Adjemian J, Lai YL, et al; National Institutes of Health Antimicrobial Resistance Outcomes Research Initiative (NIH–ARORI). Difficult-to-Treat Resistance in Gram-negative Bacteremia at 173 US Hospitals: Retrospective Cohort Analysis of Prevalence, Predictors, and Outcome of Resistance to All First-line Agents. *Clin Infect Dis 2018* 28;67:1803-14.

2. Centers for Disease Control and Prevention. Carbapenem-resistant Enterobacterales (CRE) Infection Control. <https://www.cdc.gov/cre/hcp/infection-control/index.html>. Published 2024. Accessed September 9, 2024.

**WHO Hand Hygiene Self-Assessment Framework 2010 (Japanese version)**

AMR Clinical Reference Center. Tools and supplies for medical professionals. <https://amr.ncgm.go.jp/medics/2-8-1.html>. Published 2024. Accessed July 17, 2024.

| **1．システム変更** | | | |
| --- | --- | --- | --- |
| **質 問** | **回 答** | **スコア** | **WHO 改善ツール**  **（文末参照）** |
| **1.1** | 使用できない | 0 | ① ② ③ |
| あなたの医療施設において、擦式アルコール | 使用できるが、製剤の効果^1^ と皮膚への安全性^2^ が証明さ | 0 |  |
| 性手指消毒薬はどの程度容易に使用できます | れていない |  |  |
| か？ |  |  |  |
|  | 特定の病棟でのみ使用できる、または常時供給されてい | 5 |  |
| **答えを一つ選んでください。** | ない（効果^1^ と皮膚への安全性^2^ が証明された製剤を使用） |  |  |
|  | 施設全体において使用可能であり、常時供給されている | 10 |  |
| （効 | 果^1^ と皮膚への安全性^2^ が証明された製剤を使用） |  |  |
|  | 施設全体において使用可能で常時供給されており、ほと |  |  |
|  | んどの病棟において point of care^3^ での使用が可能で  ある（効果^1^ と皮膚への安全性^2^ が証明された製剤を使用） | 30 |  |
|  | 施設全体において使用可能で常時供給されており、各 |  |  |
|  | point of care^3^ での使用が可能である（効果^1^ と皮膚へ  の安全性^2^ が証明された製剤を使用） | 50 |  |
| **1.2**  シンク（手洗い場）：病床比は？  **答えを一つ選んでください。** | 1:10 未満 | 0 | ① ③ |
|  | ほとんどの病棟において少なくとも 1:10 | 5 |  |
|  | 施設全体において少なくとも 1:10  隔離病室および集中治療室では 1:1 | 10 |  |
| **1.3**  清潔な流水^4^ が常に供給されていますか？ | いいえ | 0 | ① ③ |
|  | はい | 10 |  |
| **1.4**  各シンクには石鹸^5^ が配置されていますか？ | いいえ | 0 | ① ③ |
|  | はい | 10 |  |
| **1.5**  各シンクには使い捨てタオル（訳注 ペーパー  タオル）が配置されていますか？ | いいえ | 0 | ① ③ |
|  | はい | 10 |  |
| **1.6** | いいえ | 0 |  |
| 手指衛生製品（擦式アルコール性手指消毒薬  等）を継続的に購入するための専用の、また |  |  | ③ |
|  | はい | 10 |  |
| は利用可能な予算がありますか？ |  |  |  |

追加質問：アクションプラン

| **質問 1.1 から 1.6 の合計スコアが 100 未満の場合に限り、この質問に答えてください。**  あなたの医療施設のインフラストラクチャー^6^  を改善する現実的な計画がありますか。 | いいえ | 0 | ③ ④ |
| --- | --- | --- | --- |
|  | はい | 5 |  |
|  | **システム変更の小計** | /100 |  |

| **2．研修および教育** | | | |
| --- | --- | --- | --- |
| **質 問** | **回 答** | **スコア** | **WHO 改善ツール**  **（文末参照）** |
| **2.1**  自施設における医療従事者の研修について | | | |
| 2.1a 自施設で医療従事者が受ける手指衛生研修^7^ の頻度は？  **答えを一つ選んでください。** | なし | 0 | ① ② ③ ④  ⑤ ⑥ ⑦ |
|  | 1 回以上 | 5 |  |
|  | 医療・看護スタッフ、または他のあらゆる専門職種に対  して年に 1 回以上の定期的研修が実施されている | 10 |  |
|  | 全ての専門職種に対して就職時およびその後の年に 1 回  以上の定期的研修が義務付けられている | 20 |  |
| 2.1b 全ての医療従業者についてこの研修の受講歴をを確認する方法はあります  か？ | いいえ | 0 |  |
|  | はい | 20 |  |
| **2.2**  次の WHO 資[料（www.who.int/gpsc/5may/tools）](http://www.who.int/gpsc/5may/tools)またはそれに準ずるものを全ての医療従事者が簡単に参照で  きる環境にありますか？ | | | ⑦ |
| 2.2 a WHO Guidelines on Hand Hygiene in Health-care: A Summary  （WHO 医療における手指衛生ガイドライ  ン：要約） | いいえ | 0 | ⑧ |
|  | はい | 5 |  |
| 2.2b WHO Hand Hygiene Technical Reference Manual（WHO 手指衛生テ  クニカルリファレンスマニュアル） | いいえ | 0 | ⑤ |
|  | はい | 5 |  |
| 2.2c WHO Hand Hygiene: Why, How and When, Brochure（WHO「手指衛  生なぜ、どのように、いつ」パンフレット） | いいえ | 0 | ⑥ |
|  | はい | 5 |  |
| 2.2d WHO Glove Use Information, Leafle（t WHO「手袋の使用について」リー  フレット） | いいえ | 0 | ⑨ |
|  | はい | 5 |  |
| **2.3**  手指衛生教育プログラムの指導者として十分なスキルを持つ専門家^8^ が医療施設内で積極的  に活動していますか？ | いいえ | 0 | ② ③ ⑤ ⑦ ⑧ |
|  | はい | 15 |  |
| **2.4**  手指衛生遵守観察者の研修および 評価 システ  ムは確立されていますか？ | いいえ | 0 |  |
|  | はい | 15 |  |
| **2.5**  手指衛生研修のために割り当てられた予算が  ありますか？ | いいえ | 0 | ⑩ ⑪ ⑫ ⑬ |
|  | はい | 10 |  |
|  | **研修および教育の小計** | /100 |  |

| **3．評価およびフィードバック** | | | | |
| --- | --- | --- | --- | --- |
| **質 問** | | **回 答** | **スコア** | **WHO 改善ツール**  **（文末参照）** |
| **3.1**  擦式アルコール性手指消毒薬、石鹸、使い捨てタオルおよび他の手指衛生のための資源の評価が定期的に（年に 1 回以上）行われて  いますか？ | | いいえ | 0 | ① ② |
|  |  | はい | 10 |  |
| **3.2**  次のテーマについて医療従事者の知識を少なくとも年 1 回は（例えば研修後に）評価していますか？ | | | | |
| 3.2a 手指衛生の適応 | | いいえ | 0 | ② ③ |
|  |  | はい | 5 |  |
| 3.2b 手指衛生の正しい手技 | | いいえ | 0 |  |
|  |  | はい | 5 |  |
| **3.3 手指衛生遵守の間接的モニタリング** | | | | |
| 3.3a 擦式アルコール性手指消毒薬の消費量を定期的に（少なくとも 3 か月に 1 回）モニタリングしていますか？ | | いいえ | 0 | ② ④ |
|  |  | はい | 5 |  |
| 3.3b 石鹸の消費量を定期的に（少なくとも 3 か月に 1 回）モニタリングしていますか？ | | いいえ | 0 |  |
|  |  | はい | 5 |  |
| 3.3c 1000 延べ患者数あたり、少なくとも 20L の擦式アルコール性手指消毒薬を消費していますか？ | | いいえ（または、測定していない） | 0 |  |
|  |  | はい | 5 |  |
| **3.4 手指衛生遵守の直接的モニタリング**  この項目は、手指衛生遵守観察者が研修を受け、施設から認定されており、また、WHO「手指衛生の 5 つのタイミング（My 5  Moments for Hand Hygiene）」または同等の方法を使用する場合に限り回答する。 | | | | |
| 3.4a どのくらいの頻度で WHO 手指衛生観察ツール（WHO Hand Hygiene Observation tool）または同等の方法を用いた手指衛生遵守の直接的モニタリングを実施していますか？  **答えを一つ選んでください。** | | 実施していない | 0 | ② ⑤ ⑥ |
|  |  | 不定期 | 5 |  |
|  |  | 年に1回 | 10 |  |
|  |  | 少なくとも 3 か月に 1 回 | 15 |  |
| 3.4b WHO 手 指 衛 生 観 察 ツ ー ル（WHO Hand Hygiene Observation tool）または同等の方法を用いた場合、自施設の手指衛生遵守率はどの程度ですか。  **答えを一つ選んでください。** | | ≦ 30% | 0 | ② ⑤ ⑦ ⑧ ⑨^9^ ⑩ |
|  |  | 31 － 40% | 5 |  |
|  |  | 41 － 50% | 10 |  |
|  |  | 51 － 60% | 15 |  |
|  |  | 61 － 70% | 20 |  |
|  |  | 71 － 80% | 25 |  |
|  |  | ≧ 81% | 30 |  |
| **3.5 フィードバック** | | | | |
| 3.5a **即時フィードバック**  手指衛生の観察を行うたびに、即時に結果を医療従事者に  **フィードバック**していますか。 | | いいえ | 0 | ② ⑪ |
|  |  | はい | 5 |  |
| 3.5b **組織的フィードバック**  経時的な傾向が示された手指衛生指標に関するデータを、下記の対象者に定期的（少なくとも 6 か月毎）にフィードバックしていま  すか？ | | | | |
| 3.5b.i 医療従事者 | | いいえ | 0 | ② ⑩ |
|  |  | はい | 7.5 |  |
| 3.5b.ii 施設の管理職 / 幹部 | | いいえ | 0 |  |
|  |  | はい | 7.5 |  |
|  | **評価およびフィードバックの小計** | | /100 |  |

| **4．職場での注意喚起** | | | |
| --- | --- | --- | --- |
| **質 問** | **回 答** | **スコア** | **WHO 改善ツール**  **（文末参照）** |
| **4.1**  次のようなポスター（または同等の内容を示すもの）が掲示されていますか。 | | | ① |
| 4.1a 手指衛生の適応を示すポスター  **答えを一つ選んでください。** | 掲示されていない | 0 | ② |
|  | 一部の病棟 / 診察室・処置室に掲示されている | 15 |  |
|  | ほとんどの病棟 / 診察室・処置室に掲示されている | 20 |  |
|  | 全ての病棟 / 診察室・処置室に掲示されている | 25 |  |
| 4.1b 正しい手指消毒の方法を示すポスター  **答えを一つ選んでください** | 掲示されていない | 0 | ③ |
|  | 一部の病棟 / 診察室・処置室に掲示されている | 5 |  |
|  | ほとんどの病棟 / 診察室・処置室に掲示されている | 10 |  |
|  | 全ての病棟 / 診察室・処置室に掲示されている | 15 |  |
| 4.1c 正しい手洗いの方法を示すポスター  **答えを一つ選んでください。** | 掲示されていない | 0 | ④ |
|  | 一部の病棟 / 診察室・処置室に掲示されている | 5 |  |
|  | ほとんどの病棟 / 診察室・処置室に掲示されている | 7.5 |  |
|  | 全ての病棟 / 診察室・処置室に掲示されている | 10 |  |
| **4.2**  どのくらいの頻度で全てのポスターについて破損の有無を確認し、必要時貼りかえる組織的な監査を実施していますか？  **答えを一つ選んでください** | 実施していない | 0 | ① |
|  | 年に 1 回以上 | 10 |  |
|  | 2 － 3 か月後ごと | 15 |  |
| **4.3**  上記以外のポスターを掲示また定期的に更新することにより手指衛生を推進しています  か？ | いいえ | 0 | ① |
|  | はい | 10 |  |
| **4.4**  病棟に手指衛生情報を記したリーフレットは  おいてありますか？ | いいえ | 0 | ① ⑤ |
|  | はい | 10 |  |
| **4.5**  施設全体で、他の方法による職場での注意喚起を行っていますか？  （手指衛生キャンペーンスクリーンセーバー、  バッジ、シール等） | いいえ | 0 | ① ⑥ |
|  | はい | 15 |  |
|  | **職場での注意喚起の小計** | /100 |  |

| **5．手指衛生のための施設の安全文化** | | | | |
| --- | --- | --- | --- | --- |
| **質 問** | | **回 答** | **スコア** | **WHO 改善ツール**  **（文末参照）** |
| **5.1**  自施設で最適な手指衛生実践の実行と推進に専念する手指衛生チーム^10^ について： | | | | ① |
| 5.1a そのようなチームが結成されていますか？ | | いいえ | 0 |  |
|  |  | はい | 5 |  |
| 5.1b チームは定期的に参集していますか？（少なくとも月に 1 回） | | いいえ | 0 |  |
|  |  | はい | 5 |  |
| 5.1c チームには手指衛生を積極的に推進するための活動※時間が確保されていますか？  ※手指衛生モニタリングの指導、新規活動の企画など | | いいえ | 0 |  |
|  |  | はい | 5 |  |
| **5.2**  次に示す施設の管理職 / 幹部は、手指衛生改善を支持する強い姿勢を明確に（書面または口頭で、大多数の医療従事  者に発信する形で）示していますか。 | | | | ① ② ③ |
| 5.2a 最高責任者（理事長 / 病院長 / 施設長） | | いいえ | 0 |  |
|  |  | はい | 10 |  |
| 5.2b 診療科長・診療部門長 | | いいえ | 0 |  |
|  |  | はい | 5 |  |
| 5.2c 看護部長 | | いいえ | 0 |  |
|  |  | はい | 5 |  |
| **5.3**  5 月 5 日の「命を救おう . 手を清潔に . 手指衛生の日」において施設全体で手指衛生を推進  するための明確な計画が立案されていますか。 | | いいえ | 0 | ① ④ |
|  |  | はい | 10 |  |
| **5.4**  全ての部門において手指衛生リーダーを特定する体制はありますか？ | | | |  |
| 5.4a 手指衛生リーダー（champion）^11^ 指名システム | | いいえ | 0 |  |
|  |  | はい | 5 |  |
| 5.4b 手指衛生のロールモデル^12^ の表彰および活用システム | | いいえ | 0 |  |
|  |  | はい | 5 |  |
| **5.5**  手指衛生推進への患者の関与について： | | | | ① ⑤ |
| 5.5a 手指衛生の重要性について患者に情報提供していますか？（例：リーフレットを  使用） | | いいえ | 0 |  |
|  |  | はい | 5 |  |
| 5.5b 患者参加の正式なプログラムに着手していますか。 | | いいえ | 0 |  |
|  |  | はい | 10 |  |
| **5.6**  自施設では、各現場における継続的な改善を支援する取り組みとして、例えば次のようなものがありますか： | | | | ① ④ |
| 5.6a 手指衛生の E- ラーニングツール | | いいえ | 0 |  |
|  |  | はい | 5 |  |
| 5.6b 毎年設定される施設が達成すべき手指衛生の目標 | | いいえ | 0 |  |
|  |  | はい | 5 |  |
| 5.6c 信頼性があり、試験済みの現場のイノベーションを施設内で共有するシステム | | いいえ | 0 |  |
|  |  | はい | 5 |  |
| 5.6d 手指衛生について定期的に触れるコミュニケーションの機会（例：施設報、臨床  会議など） | | いいえ | 0 |  |
|  |  | はい | 5 |  |
| 5.6e 個人の説明責任（アカウンタビリティ）システム^13^ | | いいえ | 0 |  |
|  |  | はい | 5 |  |
| 5.6f 新規雇用者のためのバディーシステム^14^ | | いいえ | 0 |  |
|  |  | はい | 5 |  |
|  | **施設の安全文化の小計** | | /100 |  |

**Infection Prevention and Control Assessment Framework (Japanese version)**

AMR Clinical Reference Center. Tools and supplies for medical professionals. <https://amr.ncgm.go.jp/medics/2-8-1.html>. Published 2024. Accessed July 17, 2024.

| **中核的要素 1：感染予防・管理（IPC）プログラム** | | |
| --- | --- | --- |
| **質問** | **回答** | **点数** |
| **1. 施設にIPCプログラムはありますか？ 3**  1つ選択してください。 | いいえ | 0 |
|  | はい、ただし、明確な目的は定められていません | 5 |
|  | はい、目的が明確に定められており、年間の活動計画も用意  されています | 10 |
| **2. IPCプログラムは、IPC専門家で構成されるIPCチームによってサポートされていますか？4**  1つ選択してください。 | いいえ | 0 |
|  | チームではなく、1名のIPC責任者のみ | 5 |
|  | はい | 10 |
| **3. IPCチームには、少なくとも1名のフルタイムのIPC専門家または同等の資格を有する者（IPC専従の看護師または医師）がいますか？**  1つ選択してください。 | いいえ、IPC専門家はいません | 0 |
|  | パートタイムのIPC専門家のみです | 2.5 |
|  | はい、250床あたり1名未満のフルタイムIPC専門家がいます | 5 |
|  | はい、250床あたり1名以上のフルタイムIPC専門家がいます | 10 |
| **4. IPCチームまたは責任者は、IPC業務に専念する時間（プロテクトされた時間）がありますか？** | いいえ | 0 |
|  | はい | 10 |
| **5. IPCチームには医師と看護師がどちらも配置されていますか?** | いいえ | 0 |
|  | はい | 10 |
| **6. IPCチームを支援するIPC委員会5が設置されていますか？** | いいえ | 0 |
|  | はい | 10 |

**7. IPC委員会には、以下の専門家グループが含まれていますか?**

| 施設管理者（理事長、最高経営責任者[CEO]、院長・施設長など） | いいえ | 0 |
| --- | --- | --- |
|  | はい | 5 |
| 臨床スタッフ代表（医師、看護師など） | いいえ | 0 |
|  | はい | 2.5 |
| 施設保全担当者（バイオセーフティ、医療廃棄物の管理者や、水と衛生環境の担当者など） | いいえ | 0 |
|  | はい | 2.5 |
| **8. IPCの目標を明確に設定していますか（とりわけ重要分野において）？**  1つ選択してください。 | いいえ | 0 |
|  | IPCの目標のみ設定しています | 2.5 |
|  | IPCの目標と測定可能なアウトカム（すなわち、改善のための適切な指標）を設定しています | 5 |
|  | IPCの目標と測定可能なアウトカムに加え、さらに今後の目標を設定しています | 10 |

**9. 施設管理者は、IPCプログラムに対する明確なコミットメントを示し、支援を行なっていますか?**

| IPCプログラムに特別に予算を割り当てている（IPCスタッフの給与を含む IPC活動のための予算の割り当てなど） | いいえ | 0 |
| --- | --- | --- |
|  | はい | 5 |
| 施設内のIPCの目標および指標に対する明確な支援を行なっている（役員 クラスのミーティング、役員による巡視、合併症および死亡に関する（M&M）カンファレンスへの参加など） | いいえ | 0 |
|  | はい | 5 |
| **10. 日常的に（施設内または施設外の）微生物検査室の支援が得られますか?**  1つ選択してください。 | いいえ | 0 |
|  | はい、ただし結果が確実に（適時かつ正確に）出せるわけではありません | 5 |
|  | はい、結果を確実に（適時かつ正確に）出せます | 10 |
| **小計点数** |  | **/100 点** |

| **中核的要素 2：感染予防・管理（IPC）ガイドライン** | | |
| --- | --- | --- |
| **質問** | **回答** | **点数** |
| **1. 施設には、ガイドラインの策定または改変のための（IPCや感染症における）専門知識・ノウハウをもった専門家がいますか?** | いいえ | 0 |
|  | はい | 7.5 |
| **2. 施設では、以下に関するガイドラインを整備していますか？** | | |
| 標準予防策（スタンダードプリコーション） | いいえ | 0 |
|  | はい | 2.5 |
| 手指衛生 | いいえ | 0 |
|  | はい | 2.5 |
| 感染経路別予防策6 | いいえ | 0 |
|  | はい | 2.5 |
| アウトブレイクへの対応と準備 | いいえ | 0 |
|  | はい | 2.5 |
| 手術部位感染症の予防7 | いいえ | 0 |
|  | はい | 2.5 |
| 血管内留置カテーテル関連血流感染症の予防 | いいえ | 0 |
|  | はい | 2.5 |
| 院内肺炎（HAP）の予防（人工呼吸器関連肺炎を含むが、これに限らない） | いいえ | 0 |
|  | はい | 2.5 |
| カテーテル関連尿路感染症の予防 | いいえ | 0 |
|  | はい | 2.5 |
| 多剤耐性（MDR）病原体の感染伝播予防 | いいえ | 0 |
|  | はい | 2.5 |
| 消毒および滅菌 | いいえ | 0 |
|  | はい | 2.5 |
| 医療従事者の保護および安全8 | いいえ | 0 |
|  | はい | 2.5 |
| 注射の安全性 | いいえ | 0 |
|  | はい | 2.5 |
| 医療廃棄物の管理 | いいえ | 0 |
|  | はい | 2.5 |
| 抗菌薬適正使用9 | いいえ | 0 |
|  | はい | 2.5 |
| **3. 施設のガイドラインは、国内外のガイドライン（存在する場合）に準拠していますか？** | いいえ | 0 |
|  | はい | 10 |
| **4. ガイドラインの実施手段は、一般的なIPC基準を維持しつつ、施設のニーズやリソースに合わせて改変されていますか10？** | いいえ | 0 |
|  | はい | 10 |

| **5. IPCスタッフに加え、現場の医療従事者はIPCガイドラインの計画と実施の両方に関与していますか？** | いいえ | 0 |
| --- | --- | --- |
|  | はい | 10 |
| **6. IPCスタッフに加え、関係者（主任医師や看護師長、病院長、医療の質・医療安全管理部など）は、IPCガイドラインの作成および改変に関与していますか？** | いいえ | 0 |
|  | はい | 7.5 |
| **7. 医療従事者は、施設に導入された新規のまたは更新されたIPCガイドラインについて、研修を受けていますか？** | いいえ | 0 |
|  | はい | 10 |
| **8. 施設では、少なくともIPCガイドラインのいくつかについて、実施状況を定期的にモニタリングしていますか？** | いいえ | 0 |
|  | はい | 10 |
| **小計点数** |  | **/100点** |

| **中核的要素 3：感染予防・管理（IPC）に関する教育と研修** | | |
| --- | --- | --- |
| **質問** | **回答** | **点数** |
| **1. IPC研修を主導するIPC(とあるいは感染症)の専門家がいますか?** | いいえ | 0 |
|  | はい | 10 |
| **2. 上記に加え、指導者や助言者（メンター）として十分なスキルを備えた IPC専任でない医療従事者（リンクナースまたは医師など）はいますか?** 1つ選択してください。 | いいえ | 0 |
|  | はい | 10 |
| **3. 医療従事者はIPCに関する研修をどのくらいの頻度で受けていますか？**  1つ選択してください。 | まったく、あるいは、ほとんど受けていません | 0 |
|  | 新入職員オリエンテーション時のみ | 5 |
|  | 新入職員オリエンテーションおよび医療従事者向けの定期的 な（少なくとも年1回）IPC研修が提供されていますが、参加は義務  ではありません | 10 |
|  | 新入職員オリエンテーションおよび定期的な（少なくとも年1  回）IPC研修が全医療従事者に義務づけられています | 15 |
| **4. 清掃員や患者ケアに直接関与する他の職員は、IPCに関する研修をどのくらいの頻度で受けていますか？**  1つ選択してください。 | まったく、あるいは、ほとんど受けていません | 0 |
|  | 新入職員オリエンテーション時のみ | 5 |
|  | 新入職員オリエンテーションおよび定期的な（少なくとも年1回）IPC研修が提供されていますが、参加は義務ではありません | 10 |
|  | 新入職員オリエンテーションおよび定期的な（少なくとも年1回）IPC研修が義務づけられています | 15 |
| **5. 事務職員は、IPCに関する一般的な研修を受けていますか?**  1つ選択してください。 | いいえ | 0 |
|  | はい | 5 |
| **6. 医療従事者およびその他の職員はどのように研修を受けていますか？**  1つ選択してください。 | 研修はありません | 0 |
|  | 文書・口頭指導・eラーニングのみ | 5 |
|  | 上記に加え、双方向性のトレーニングセッション（シミュレーション・ベッドサイド研修など） | 10 |
| **7. 研修プログラムの効果について定期的に評価していますか（手指衛生監査、知識に関するその他のチェックなど)？**  1つ選択してください。 | いいえ | 0 |
|  | はい、ただし定期的ではありません | 5 |
|  | はい、定期的に（少なくとも年1回）実施しています | 10 |
| **8. IPC研修は、日常診療や他の専門分野の研修に統合されていますか（例：外科医の研修にIPCの観点が含まれる）？**  1つ選択してください。 | いいえ | 0 |
|  | はい、一部の分野で | 5 |
|  | はい、すべての分野で | 10 |
| **9. 医療関連感染症を最小限に抑えるため、患者または家族に対するIPC研修がありますか？**  （免疫抑制患者、侵襲的デバイスを使用している患者、多剤耐性感染症を有する患者など） | いいえ | 0 |
|  | はい | 5 |
| **10. スタッフに対して継続的な人材開発・教育が提供されていますか（定期的な学会や研修への参加など）?** | いいえ | 0 |
|  | はい | 10 |
| **小計点数** |  | **/100 点** |

| **中核的要素 4：医療関連感染症（HAI）サーベイランス** | | |
| --- | --- | --- |
| **質問** | **回答** | **点数** |

**サーベイランス実施体制**

| **1. サーベイランスは施設のIPCプログラムに含まれていますか？** | いいえ | 0 |
| --- | --- | --- |
|  | はい | 5 |
| **2. サーベイランス業務を担当するスタッフはいますか?** | いいえ | 0 |
|  | はい | 5 |
| **3. サーベイランス業務を担当するスタッフは、疫学、サーベイランス、IPCに関してトレーニングを受けていますか（サーベイランスの手法、データ管理と解釈など）？** | いいえ | 0 |
|  | はい | 5 |
| **4. サーベイランスを実施するにあたり、ITサポートが整備されていますか**  **（設備、モバイル技術、電子カルテなど）？** | いいえ | 0 |
|  | はい | 5 |

**サーベイランスの優先順位（施設での医療ケアの性質・特徴によって決定される）**

| **5. 地域の状況に応じて、サーベイランスの対象となる医療関連感染症の優先順位付けを行っていますか（すなわち、施設における罹患・死亡の主要原因となる感染症を特定している）？11** | いいえ | 0 |
| --- | --- | --- |
|  | はい | 5 |
| **6. 以下のサーベイランスを実施していますか？** | | |
| 手術部位感染症12 | いいえ | 0 |
|  | はい | 2.5 |
| デバイス関連感染症（カテーテル関連尿路感染症、中心静脈ライン関連血流感染症、末梢静脈ライン関連血流感染症、人工呼吸器関連肺炎など） | いいえ | 0 |
|  | はい | 2.5 |
| 臨床的に定義された感染症（微生物学検査が実施されず臨床徴候または症状のみに基づく） | いいえ | 0 |
|  | はい | 2.5 |
| 地域の疫学的状況に応じた多剤耐性 13病原体による保菌または感染症 | いいえ | 0 |
|  | はい | 2.5 |
| 流行性の観点から地域で優先される感染症（ノロウイルス、インフルエンザ、結核[TB]、重症急性呼吸器症候群[SARS]、エボラウイルス、ラッサ熱など） | いいえ | 0 |
|  | はい | 2.5 |
| 易感染患者群（新生児、集中治療室患者、免疫不全患者、熱傷患者など）における感染症14 | いいえ | 0 |
|  | はい | 2.5 |
| 臨床、検査、その他の医療環境で医療従事者に影響を及ぼす可能性のある感染症（B型・C型肝炎、ヒト免疫不全ウイルス[HIV]、インフルエンザなど） | いいえ | 0 |
|  | はい | 2.5 |
| **7. サーベイランスが現在のニーズおよび施設における優先事項に沿って行われているか、定期的に評価していますか？11** | いいえ | 0 |
|  | はい | 5 |

| **調査方法** | | |
| --- | --- | --- |
| **8. 信頼できるサーベイランスの症例定義（例えばCDC NHSNやECDC15など国際的な定義）を使用していますか？あるいは、変更して運用される場合は、エビデンスに基づく改変プロセスおよび専門家のコンサルテーションを通じて変更/運用していますか？** | いいえ | 0 |
|  | はい | 5 |
| **9. 国際的なサーベイランスプロトコル（CDC NHSNやECDCなど）に則って標準化されたデータ収集方法（積極的前向きサーベイランスなど）を使用していますか？ あるいは、変更して運用される場合は、エビデンスに基づく改変プロセスおよび専門家のコンサルテーションを通じて変更/運用していますか?** | いいえ | 0 |
|  | はい | 5 |
| **10. データの質を定期的に担保するための仕組みが整備されていますか（症例報告書の評価、微生物学検査結果の確認、サーベイランスにおける分母となる数・定義の決定など）？** | いいえ | 0 |
|  | はい | 5 |
| **11. サーベイランスをサポートするため、微生物学検査を含む十分な検査体制がありますか?**  1つ選択してください。 | いいえ | 0 |
|  | グラム陽性菌/陰性菌を判別することができます。しかし、病原体を特定することはできません。 | 2.5 |
|  | 病原体（分離同定など）を適時かつ正確に特定することができます。 | 5 |
|  | 病原体および薬剤耐性のパターン（すなわち、感受性）を適時かつ正確に特定することができます | 10 |
| **情報分析と通知／データ使用、連携、ガバナンス** | | |
| **12. サーベイランスデータを用いて個々の病棟や施設に合わせてカスタマイズしたIPC改善計画を作成していますか？** | いいえ | 0 |
|  | はい | 5 |
| **13. 薬剤耐性の状況を定期的（年4回／年2回／年1回など）に分析していますか？** | いいえ | 0 |
|  | はい | 5 |
| **14. 最新のサーベイランス情報を以下の者に対し、定期的（年4回／年2回／年1回など）にフィードバックしていますか?** | | |
| 現場の医療従事者（医師・看護師） | いいえ | 0 |
|  | はい | 2.5 |
| 部門責任者 | いいえ | 0 |
|  | はい | 2.5 |
| IPC委員会 | いいえ | 0 |
|  | はい | 2.5 |
| 非臨床の管理部門・経営陣（最高経営責任者・最高財務責任者） | いいえ | 0 |
|  | はい | 2.5 |
| **15. 最新のサーベイランス情報を（少なくとも年1回）どのようにフィードバックしていますか?**  1つ選択してください。 | フィードバックは行っていません | 0 |
|  | 文書・口頭による情報提供のみ | 2.5 |
|  | プレゼンテーションおよび双方向型の問題志向型解決法による | 7.5 |
| **小計点数** |  | **/100 点** |

| **中核的要素 5：感染予防・管理（IPC）の実践に関する複合的改善戦略16** | | |
| --- | --- | --- |
| **質問** | **回答** | **点数** |
| **1. IPCを実践するために、複合的改善戦略16を用いていますか?** | いいえ | 0 |
|  | はい | 15 |
| **2. 複合的改善戦略には以下の構成要素のいずれか、またはすべてが含まれていますか？**  1つの要素につき（最も該当する）1つの回答を選択してください。 | **システム変更** |  |
|  | 複合的改善戦略に含まれていません | 0 |
|  | 必要なインフラと継続的な物品を確保するための取り組みが整備されていま  す | 5 |
|  | 上記に加え、人間工学17と使いやすさ（中心静脈カテーテルのセットとトレーの最適な配置など）を考慮しています | 10 |
|  | **研修および教育** |  |
|  | 複合的改善戦略に含まれていません | 0 |
|  | 文書・口頭指導・eラーニングのみ | 5 |
|  | 上記に加え、双方向性のトレーニングセッション（シミュレーション・ベッドサイド研修など） | 10 |
|  | **モニタリング（評価）とフィードバック** |  |
|  | 複合的改善戦略に含まれていません | 0 |
|  | プロセスまたは結果（アウトカム）指標のモニタリング（手指衛生またはカテーテルの取り扱いの監査など） | 5 |
|  | 上記に加え、モニタリング結果を医療従事者および関係者に適宜フィードバック | 10 |
|  | **コミュニケーションと注意喚起** |  |
|  | 複合的改善戦略に含まれていません | 0 |
|  | 取り組みを促すための注意喚起、ポスター、またはその他の支援・意識向上ツール | 5 |
|  | 上記に加え、部門を超えてコミュニケーションを改善するための付加的な手段・取り組み（定期的な症例検討会やフィードバックのための回診など） | 10 |
|  | **医療安全に対する組織風土・文化の強化** |  |
|  | 複合的改善戦略に含まれていません | 0 |
|  | 部門責任者はロールモデルとなり、適応アプローチを取り18 、IPC、医療安全および医療の質を改善する組織文化の強化に取り組んでいます | 5 |
|  | 上記に加え、チームおよび個人には、取り組みに対する当事者意識を持ってもらうよう、工夫がなされています（参加型回診など） | 10 |

| **3. IPCの複合的改善戦略を実施するために多職種横断的なチームがありますか？** | いいえ | 0 |
| --- | --- | --- |
|  | はい | 15 |
| **4. IPCの複合的改善戦略の策定・推進のため、医療の質改善や医療安全部門のスタッフと定期的に連携していますか?** | いいえ | 0 |
|  | はい | 10 |
| **5. 戦略にはバンドル19やチェックリストが含まれていますか？** | いいえ | 0 |
|  | はい | 10 |
| **小計点数** |  | **/100 点** |

| **中核的要素 6：IPC実践のモニタリング・監査およびフィードバック** | | |
| --- | --- | --- |
| **質問** | **回答** | **点数** |
| **1. IPC実践のモニタリング・監査およびフィードバックを担当するため、トレーニングを受けた職員がいますか?** | いいえ | 0 |
|  | はい | 10 |
| **2. 目標、活動内容（体系的にデータを収集するためのツールなど）が明確に定められたモニタリング計画がありますか?** | いいえ | 0 |
|  | はい | 7.5 |
| **3. どのプロセスおよび指標をモニターしていますか?**  該当するものすべてにチェックを入れてください | なし | 0 |
|  | 手指衛生遵守率・コンプライアンス（WHO手指衛生観察ツール20または同等のものを使用） | 5 |
|  | 血管内留置カテーテルの挿入・ケア | 5 |
|  | 創部ドレッシングの交換 | 5 |
|  | 多剤耐性菌（MDRO）の伝播を防止するための感染経路別予防策および隔離 | 5 |
|  | 病棟・施設の清掃 | 5 |
|  | 医療機器の消毒および滅菌 | 5 |
|  | 擦式アルコール製剤または石鹸の使用量 | 5 |
|  | 抗菌薬の使用量 | 5 |
|  | 医療廃棄物の管理 | 5 |
| **4. WHO手指衛生自己評価フレームワーク21 はどのくらいの頻度で使われていますか？**  1つ選択してください。 | 使われていません | 0 |
|  | ときどき使われていますが、定期的なスケジュールが組まれて  いるわけではありません | 2.5 |
|  | 少なくとも年1回実施 | 5 |
| **5. IPC活動や成果に関する監査の結果を報告・フィードバックしていますか（手指衛生遵守率データやその他のプロセスに関するフィードバックなど）？**  該当するものすべてにチェックを入れてください | 行なっていません | 0 |
|  | IPCチーム内で | 2.5 |
|  | 監査対象の部門責任者に対して | 2.5 |
|  | 現場の医療従事者に対して | 2.5 |
|  | IPC委員会または医療の質委員会、または同等の委員会で | 2.5 |
|  | 施設管理部門および経営陣に対して | 2.5 |
| **6. モニタリングデータの報告は定期的に（少なくとも年1回）行われていますか？** | いいえ | 0 |
|  | はい | 10 |
| **7. IPCのプロセスおよび指標に関するモニタリングならびにフィードバックは、改善および行動変容を目的とし、「非難しない」組織文化の中で行われていますか？** | いいえ | 0 |
|  | はい | 5 |
| **8.施設における医療安全の文化を評価していますか？**  **（HSOPSC、SAQ、PSCHO、HSC22 など他の調査法を使用して）** | いいえ | 0 |
|  | はい | 5 |
| **小計点数** |  | **/100 点** |

| **中核的要素 7：仕事量、人員配置およびベッド占有率 23** | | |
| --- | --- | --- |
| **質問** | **回答** | **点数** |
| **人員配置** | | |
| **1. 国の基準や、WHO人員配置の必要性評価に用いる作業負荷の指標 24といった標準的評価ツールを用いて、作業負荷に応じた適切な人員配置レベルを評価していますか？** | いいえ | 0 |
|  | はい | 5 |
| **2. 施設内の医療従事者と患者との比率25は（WHOや国が定めた）規定された比率を維持していますか？**  1つ選択してください。 | いいえ | 0 |
|  | 50%未満の部署で維持 | 5 |
|  | 50%以上の部署で維持 | 10 |
|  | 施設全体で維持 | 15 |
| **3. 人員配置レベルが低すぎる場合、人員配置の必要性評価の結果に基づき対応する仕組みがありますか？** | いいえ | 0 |
|  | はい | 10 |
| **ベッド占有率** | | |
| **4. 病棟の設計は、ベッド占有率に関する国際基準26に準じていますか？**  1つ選択してください。 | いいえ | 0 |
|  | はい、ただし特定の部門に限定 | 5 |
|  | はい、（救急科、小児科を含む）すべての部門において | 15 |
| **5. ベッド占有率は1床あたり患者1名に維持されていますか？**  1つ選択してください。 | いいえ | 0 |
|  | はい、ただし特定の部門に限定 | 5 |
|  | はい、（救急科、小児科を含む）すべての部門において | 15 |
| **6. 患者が（救急科を含め）病室外の廊下に設置されたベッドに割り当てられることはありますか？**  1つ選択してください。 | 週2回以上 | 0 |
|  | 週2回未満 | 5 |
|  | いいえ、ありません | 15 |
| **7. 患者のベッド同士の間に1メートルを超える十分なスペースを確保していますか？**  1つ選択してください。 | いいえ | 0 |
|  | はい、ただし特定の部門に限定 | 5 |
|  | はい、（救急科、小児科を含む）すべての部門において | 15 |
| **8. 適切なベッド占有率を超えている場合、対応するための仕組みがありますか？**  1つ選択してください。 | いいえ | 0 |
|  | 対応するのは部門長の責任 | 5 |
|  | 対応するのは病院の管理部門・経営陣 | 10 |
| **小計点数** |  | **/100 点** |

| **中核的要素 8：施設レベルでのIPCに備えたインフラ、資材および設備27** | | |
| --- | --- | --- |
| **質問** | **回答** | **点数** |
| **水** | | |
| **1. すべての用途（手洗い、飲料水、個人衛生、医療活動、滅菌、除染、清掃、洗濯など）に常に十分な量の水が利用可能ですか？**  1つ選択してください。 | 利用できるのは週平均5日未満 | 0 |
|  | 1週間で平均5日以上、または毎日利用可能ですが、十分な量ではありません | 2.5 |
|  | 毎日かつ十分な量が利用可能 | 7.5 |
| **2. 信頼できる安全な飲料水があり、職員・患者・家族が常時すべての場所・病棟で利用できますか?**  1つ選択してください。 | いいえ | 0 |
|  | ときどき、または一部の場所でのみ利用できます。または、すべ  ての人が利用できるわけではありません | 2.5 |
|  | はい、常時かつすべての病棟ですべての人が利用可能です | 7.5 |
| **手指衛生と衛生設備** | | |
| **3. 手指衛生の設備・物品（擦式アルコール製剤、または石鹸、水および清潔な使い捨てタオルなど）が必要なすべての場面で利用可能ですか？**  1つ選択してください。 | いいえ | 0 |
|  | 手指衛生を行う設備はありますが、物品は確実に使用できるわ  けではありません | 2.5 |
|  | 設備に加え、物品も常に利用可能です | 7.5 |
| **4. 外来患者用に4基以上、もしくは入院患者用に20名につき1基以上のトイレまたは改良型仮設トイレ28が利用可能ですか？**  1つ選択してください。 | 利用可能なトイレの数が必要数より不足 | 0 |
|  | 十分な数が設置されていますが、そのすべてが利用できるわけ  ではありません | 2.5 |
|  | 十分な数が設置されており、かつ利用可能です | 7.5 |
| **電源、換気、清掃** | | |
| **5. あらゆる用途のために、日中および夜間に十分なエネルギー・電力供給がありますか？（水の汲み上げや煮沸、滅菌および除染、焼却または代替処理技術、電子医療機器、照明など）**  1つ選択してください。 | いいえ | 0 |
|  | 時々、または一部区域に限定して | 2.5 |
|  | 常に、かつすべての区域において利用可能 | 5 |
| **6. 患者ケア区域内では、環境換気（自然または機械換気29）が機能していますか？** | いいえ | 0 |
|  | はい | 5 |
| **7. 清掃員が毎日署名する床や水平面の清掃記録がありますか？**  1つ選択してください。 | ありません | 0 |
|  | 記録は存在しますが、完全には記入されておらず、毎日署名されていない、または更新されていません | 2.5 |
|  | はい、毎日記録・署名されています | 5 |
| **8. 適切かつ十分なメンテナンスがされている清掃用品（洗剤、モップ、バケツなど）がありますか？**1つ選択してください。 | ありません | 0 |
|  | 配備されていますが不十分なメンテナンスです | 2.5 |
|  | 配備され、かつ、しっかりとメンテナンスされています | 5 |

| **医療現場における患者の配置および個人用保護具（PPE）** | | |
| --- | --- | --- |
| **9. 結核、麻疹、コレラ、エボラウイルス感染症、SARSなどの患者を専用に隔離する部屋の数が不十分な場合、一人部屋または同様の病原体を持つ患者を近くに集めて収容する（コホーティング）30ための部屋が用意され ていますか？31**  1つ選択してください。 | いいえ | 0 |
|  | 一人部屋はありませんが、コホーティングに適した部屋はあります | 2.5 |
|  | はい、一人部屋があります | 7.5 |
| **10. すべての医療従事者があらゆる場面で使用するために、十分な数の個人用保護具（PPE）32が常に用意されていますか？**  1つ選択してください。 | いいえ | 0 |
|  | はい、ただし、十分な数を継続的に利用できるわけではありま  せん | 2.5 |
|  | はい、十分な数を継続的に利用することが可能です | 7.5 |
| **医療廃棄物の管理と下水** | | |
| **11. 廃棄物が発生するすべての場所の近くに、非感染性（一般）廃棄物、感染性廃棄物、および鋭利廃棄物を廃棄できる収集容器が設置されていますか？**  1つ選択してください。 | ゴミ箱または個別の鋭利廃棄物収集容器が設置されていませ  ん | 0 |
|  | 個別のゴミ箱は設置されていますが、①蓋がない・または容量の4分の3以上が一杯になっている、②（3種類ではなく）2種類のゴミ箱のみ配置されている、または③廃棄物が発生する場所のすべ  てではなく一部の場所のみに設置されています | 2.5 |
|  | はい | 5 |
| **12. 非感染性廃棄物（非危険物・一般廃棄物）の処理のため、施設内にフェンスで囲まれた廃棄場所、または市町村や業者による廃棄物回収がありますか？**  1つ選択してください。 | いいえ | 0 |
|  | 施設内に廃棄場所はあるが、大きさが不十分、廃棄場所があふ  れている、フェンスやロックがない、または廃棄物回収が不定期 | 2.5 |
|  | はい | 5 |
| **13. 感染性廃棄物および鋭利廃棄物を処理するための焼却炉または代替処理技術（オートクレーブなど）は敷地内または敷地外に存在し、専門の廃棄物管理部門・業者によって管理されていますか？**  1つ選択してください。 | いいえ | 0 |
|  | 整備されていますが、機能していません | 2.5 |
|  | はい | 5 |
| **14. 排水処理システム（下水処理タンクとそれに続く排水溜めなど）が（敷地内または敷地外に）整備されており、確実に機能していますか？**  1つ選択してください。 | いいえ | 0 |
|  | はい、ただし確実には機能していません | 2.5 |
|  | はい、整備されており、かつ確実に機能しています | 5 |
| **除染・滅菌** | | |
| **15. 医療機器およびその他の物品・装置の除染および滅菌を行うために、敷地内または敷地外に、専門の除染管理部門・業者によって管理されている専用除染区域や滅菌設備がありますか?**  1つ選択してください。 | いいえ | 0 |
|  | はい、ただし確実には機能していません | 2.5 |
|  | はい、整備されており、かつ確実に機能しています | 5 |
| **16.すぐに使用できるように、滅菌および消毒済みの物品を確実に用意していますか?**  1つ選択してください。 | 使用できるのは週平均5日未満です | 0 |
|  | 週平均5日以上または毎日利用可能ですが、十分な数は用意されていません | 2.5 |
|  | はい、毎日、かつ十分な数を用意しています | 5 |
| **17. 必要に応じて使い捨て物品が用意されていますか？（安全装置付き注射針、診察用手袋など）**  1つ選択してください。 | いいえ | 0 |
|  | 常に用意されているわけではありません | 2.5 |
|  | はい、常に用意されています | 5 |
| **小計点数** |  | **/100 点** |

**解釈：3段階のプロセス**

**1. 点数の合計**

|  | **点数** |
| --- | --- |
| **セクション（中核的要素）** | **小計** |
| 1. IPCプログラム |  |
| 2. IPCガイドライン |  |
| 3. IPC教育・研修 |  |
| 4. HAIサーベイランス |  |
| 5. 複合的改善戦略 |  |
| 6. モニタリング・監査およびフィードバック |  |
| 7. 仕事量、人員配置およびベッド占有率 |  |
| 8. インフラ、資材および設備 |  |
| **合計点** | **/800 点** |

**2. ステップ1の合計点数を用いて、該当する「IPCレベル」を決定します。**

| **合計点**（範囲）) | **IPCレベル** |
| --- | --- |
| 0–200 | 不十分 |
| 201–400 | 最低限 |
| 401–600 | 中等度 |
| 601–800 | 十分 |

**3. フレームワークの結果を振り返り、行動計画を策定する**

今回の評価で改善が必要だと特定された領域を見直し、これに対応するための行動計画を策定してください。この作業に取り組むにあたり、感染予防・管理プログラム中核的要素に関するWHOガイドラインの実施を支援する暫定実践マニュアル2 を参照してください。世界中から集められた指針、テンプレート、ヒント、事例や、関連するIPC改善ツールのリストが記載されています。将来再評価した時に比較できるよう、今回の評価内容のコピーを保管してください。

**WHO Hand Hygiene Self-Assessment Framework 2010**

World Health Organization. Hand hygiene Monitoring tools. <https://www.who.int/teams/integrated-health-services/infection-prevention-control/hand-hygiene/monitoring-tools>. Published 2010. Accessed July 26, 2024.

| **1. System Change** | | | |
| --- | --- | --- | --- |
| **Question** | **Answer** | **Score** | **WHO improvement tools** |
| **1.1**  How easily available is alcohol-based handrub in your health-care facility?  **Choose one answer** | Not available | 0 | 🡢 Ward Infrastructure Survey  🡢 Protocol for Evaluation of Tolerability and Acceptability of Alcohol-based Handrub  in Use or Planned to be Introduced:Method 1  🡢 Guide to Implementation II.1 |
|  | Available, but efficacy1 and tolerability2 have not been proven | 0 |  |
|  | Available only in some wards or in discontinuous supply (with efficacy1 and tolerability2 proven) | 5 |  |
|  | Available facility-wide with continuous supply (with efficacy1 and tolerability2 proven) | 10 |  |
|  | Available facility-wide with continuous supply, and at the point of care3 in the majority of wards  (with efficacy1 and tolerability2 proven) | 30 |  |
|  | Available facility-wide with continuous supply at each point of care3 (with efficacy1 and tolerability2 proven) | 50 |  |
| **1.2**  What is the sink:bed ratio?  **Choose one answer** | Less than 1:10 | 0 | 🡢 Ward Infrastructure Survey  🡢 Guide to Implementation II.1 |
|  | At least 1:10 in most wards | 5 |  |
|  | At least 1:10 facility-wide and 1:1 in isolation rooms and in intensive care units | 10 |  |
| **1.3**  Is there a continuous supply of clean, running water4? | No | 0 | 🡢 Ward Infrastructure Survey  🡢 Guide to Implementation II.1 |
|  | Yes | 10 |  |
| **1.4**  Is soap5 available at each sink? | No | 0 | 🡢 Ward Infrastructure Survey  🡢 Guide to Implementation II.1 |
|  | Yes | 10 |  |
| **1.5**  Are single-use towels available at each sink? | No | 0 | 🡢 Ward Infrastructure Survey  🡢 Guide to Implementation II.1 |
|  | Yes | 10 |  |
| **1.6**  Is there dedicated/available budget for the continuous procurement of hand hygiene products (e.g. alcohol-based handrubs)? | No | 0 | 🡢 Guide to Implementation II.1 |
|  | Yes | 10 |  |

Extra Question: Action plan

| **Answer this question ONLY if you scored less than 100 for questions 1.1 to 1.6:**  Is there realistic plan in place to improve the infrastructure6 in your health-care facility? | No | 0 | 🡢 Alcohol-based Handrub Planning and Costing Tool  🡢 Guide to Local Production: WHO-recommended Handrub Formulations  🡢 Guide to Implementation II.1 |
| --- | --- | --- | --- |
|  | Yes | 5 |  |
|  | **System Change subtotal** | **/100** |  |

| **2. Training and Education** | | | |
| --- | --- | --- | --- |
| **Question** | **Answer** | **Score** | **WHO improvement tools** |
| **2.1**  Regarding training of health-care workers in your facility: | | | |
| 2.1a How frequently do health-care workers receive training regarding hand hygiene7 in your facility?  **Choose one answer** | Never | 0 | 🡢 Slides for Education Session for Trainers, Observers and Health-care Workers  🡢 Hand Hygiene Training Films  🡢 Slides Accompanying the Training Films  🡢 Slides for the Hand Hygiene Co-ordinator  🡢 Hand Hygiene Technical Reference Manual  🡢 Hand Hygiene Why, How and When Brochure  🡢 Guide to Implementation II.2 |
|  | At least once | 5 |  |
|  | Regular training for medical and nursing staff, or all professional categories (at least annually) | 10 |  |
|  | Mandatory training for all professional categories at commencement of employment, then ongoing regular training (at least annually) | 20 |  |
| 2.1b Is a process in place to confirm that all health-care workers complete this training? | No | 0 |  |
|  | Yes | 20 |  |
| **2.2**  Are the following WHO documents (available at [www.who.int/gpsc/5may/tools),](http://www.who.int/gpsc/5may/tools)) or similar local adaptations, easily available to all health-care workers? | | | 🡢 Guide to Implementation II.2 |
| 2.2a The ‘WHO Guidelines on Hand Hygiene in Health-care: A Summary’ | No | 0 | 🡢 WHO Guidelines on Hand Hygiene in Health Care: A Summary |
|  | Yes | 5 |  |
| 2.2b The WHO ‘Hand Hygiene Technical Reference Manual’ | No | 0 | 🡢 Hand Hygiene Technical Reference Manual |
|  | Yes | 5 |  |
| 2.2c The WHO ‘Hand Hygiene: Why, How and When’ Brochure | No | 0 | 🡢 Hand Hygiene Why, How and When Brochure |
|  | Yes | 5 |  |
| 2.2d The WHO ‘Glove Use Information’ Leaflet | No | 0 | 🡢 Glove Use Information Leaflet |
|  | Yes | 5 |  |
| **2.3**  Is a professional with adequate skills8 to serve as trainer for hand hygiene  educational programmes active within the health-care facility? | No | 0 | 🡢 WHO Guidelines on Hand Hygiene in Health Care  🡢 Hand Hygiene Technical Reference Manual  🡢 Hand Hygiene Training Films  🡢Slides Accompanying the Training Films  🡢 Guide to Implementation II.2 |
|  | Yes | 15 |  |
| **2.4**  Is a system in place for training and validation of hand hygiene compliance observers? | No | 0 |  |
|  | Yes | 15 |  |
| **2.5**  Is there is a dedicated budget that allows for hand hygiene training? | No | 0 | 🡢 Template Letter to Advocate Hand Hygiene to Managers  🡢 Template Letter to communicate Hand Hygiene Initiatives to Managers  🡢 Template Action Plan  🡢 Guide to Implementation II.2 and III.1 (page 33) |
|  | Yes | 10 |  |
|  | **Training and Education subtotal** | **/100** |  |

| **3. Evaluation and Feedback** | | | | |
| --- | --- | --- | --- | --- |
| **Question** | | **Answer** | **Score** | **WHO improvement tools** |
| **3.1**  Are regular (at least annual) ward-based audits undertaken to assess the availability of handrub, soap, single use towels and other hand hygiene resources? | | No | 0 | 🡢 Ward Infrastructure Survey  🡢 Guide to Implementation II.3 |
|  |  | Yes | 10 |  |
| **3.2**  Is health care worker knowledge of the following topics assessed at least annually (e.g. after education sessions)? | | | | |
| 3.2a. The indications for hand hygiene | | No | 0 | 🡢 Hand Hygiene Knowledge Questionnaire for Health-Care Workers  🡢 Guide to Implementation II.3 |
|  |  | Yes | 5 |  |
| 3.2b. The correct technique for hand hygiene | | No | 0 |  |
|  |  | Yes | 5 |  |
| **3.3 Indirect Monitoring of Hand Hygiene Compliance** | | | | |
| 3.3a Is consumption of alcohol-based handrub monitored regularly (at least every 3 months)? | | No | 0 | 🡢 Soap/Handrub Consumption Survey  🡢 Guide to Implementation II.3 |
|  |  | Yes | 5 |  |
| 3.3b Is consumption of soap monitored regularly (at least every 3 months)? | | No | 0 |  |
|  |  | Yes | 5 |  |
| 3.3c Is alcohol based handrub consumption at least 20L per 1000 patient-days? | | No (or not measured) | 0 |  |
|  |  | Yes | 5 |  |
| **3.4 Direct Monitoring of Hand Hygiene Compliance**  Only complete section 3.4 if hand hygiene compliance observers in your facility have been trained and validated and utilise the WHO ‘My 5 Moments for Hand Hygiene’ (or similar) methodology | | | | |
| 3.4a How frequently is direct observation of hand hygiene compliance performed using the WHO Hand Hygiene Observation tool (or similar technique)? | | Never | 0 | 🡢 WHO Hand Hygiene Observation form  🡢 Hand Hygiene Technical Reference Manual |
|  |  | Irregularly | 5 |  |
|  |  | Annually | 10 |  |
| **Choose one answer** | |  |  | 🡢 Guide to Implementation II.3 |
|  |  | Every 3 months or more often | 15 |  |
| 3.4b What is the overall hand hygiene compliance rate | | ≤ 30% | 0 | 🡢 Guide to Implementation II.3  🡢 Observation form  🡢 Data Entry Analysis tools  🡢 Instructions for Data Entry and Analysis  🡢 Epi InfoTM software9  🡢 Data Summary Report Framework |
| according to the WHO Hand Hygiene Observation tool (or | |  |  |  |
|  |  | 31 – 40% | 5 |  |
| similar technique) in your facility? | |  |  |  |
|  |  | 41 – 50% | 10 |  |
| **Choose one answer** | |  |  |  |
|  |  | 51 – 60% | 15 |  |
|  | | 61 – 70% | 20 |  |
|  | | 71 – 80% | 25 |  |
|  | | ≥ 81% | 30 |  |
| **3.5 Feedback** | | | | |
| 3.5a **Immediate feedback**  Is immediate feedback given to health-care workers at the end of each hand hygiene compliance observation session? | | No | 0 | 🡢 Guide to Implementation II.3  🡢 Observation and Basic Compliance Calculation forms |
|  |  | Yes | 5 |  |
| 3.5b **Systematic feedback**  Is regular (at least 6 monthly) feedback of data related to hand hygiene indicators with demonstration of trends over time given to: | | | | 🡢 Data Summary Report Framework  🡢 Guide to Implementation II.3 |
| 3.5b.i Health-care workers? | | No | 0 |  |
|  |  | Yes | 7.5 |  |
| 3.5b.ii Facility leadership? | | No | 0 |  |
|  |  | Yes | 7.5 |  |
|  | **Evaluation and Feedback subtotal** | | **/100** |  |

| **4. Reminders in the Workplace** | | | |
| --- | --- | --- | --- |
| **Question** | **Answer** | **Score** | **WHO improvement tools** |
| **4.1**  Are the following posters (or locally produced equivalent with similar content) displayed? | | | 🡢 Guide to Implementation II.4 |
| 4.1a Poster explaining the indications for hand hygiene  **Choose one answer** | Not displayed | 0 | 🡢 Your 5 Moments for Hand Hygiene (Poster) |
|  | Displayed in some wards/treatment areas | 15 |  |
|  | Displayed in most wards/treatment areas | 20 |  |
|  | Displayed in all wards/treatment areas | 25 |  |
| 4.1b Poster explaining the correct use of handrub  **Choose one answer** | Not displayed | 0 | 🡢 How to Handrub (Poster) |
|  | Displayed in some wards/treatment areas | 5 |  |
|  | Displayed in most wards/treatment areas | 10 |  |
|  | Displayed in all wards/treatment areas | 15 |  |
| 4.1c Poster explaining correct hand- washing technique  **Choose one answer** | Not displayed | 0 | 🡢 How to Handwash (Poster) |
|  | Displayed in some wards/treatment areas | 5 |  |
|  | Displayed in most wards/treatment areas | 7.5 |  |
|  | Displayed at every sink in all wards/treatment areas | 10 |  |
| **4.2**  How frequently does a systematic audit of all posters for evidence of damage occur, with replacement as required?  **Choose one answer** | Never | 0 | 🡢 Guide to Implementation II.4 |
|  | At least annually | 10 |  |
|  | Every 2-3 months | 15 |  |
| **4.3**  Is hand hygiene promotion undertaken by displaying and regularly updating posters other than those mentioned above? | No | 0 | 🡢 Guide to Implementation II.4 |
|  | Yes | 10 |  |
| **4.4**  Are hand hygiene information leaflets available on wards? | No | 0 | 🡢 Hand Hygiene: When and How Leaflet  🡢 Guide to Implementation II.4 |
|  | Yes | 10 |  |
| **4.5**  Are other workplace reminders located throughout the facility?  (e.g. hand hygiene campaign screensavers, badges, stickers, etc) | No | 0 | 🡢 SAVE LIVES: Clean Your Hands Screensaver  🡢 Guide to Implementation II.4 |
|  | Yes | 15 |  |
|  | **Reminders in the Workplace subtotal** | **/100** |  |

| **5. Institutional Safety Climate for Hand Hygiene** | | | | |
| --- | --- | --- | --- | --- |
| **Question** | | **Answer** | **Score** | **WHO improvement tools** |
| **5.1**  With regard to a hand hygiene team10 that is dedicated to the promotion and implementation of optimal hand  hygiene practice in your facility: | | | | 🡢 Guide to Implementation II.5 |
| 5.1a Is such a team established? | | No | 0 |  |
|  |  | Yes | 5 |  |
| 5.1b Does this team meet on a regular basis (at least monthly)? | | No | 0 |  |
|  |  | Yes | 5 |  |
| 5.1c Does this team have dedicated time to conduct active hand hygiene promotion? (e.g. teaching monitoring hand hygiene performance, organizing new activities) | | No | 0 |  |
|  |  | Yes | 5 |  |
| **5.2**  Have the following members of the facility leadership made a clear commitment to support hand hygiene improvement?  (e.g. a written or verbal commitment to hand hygiene promotion received by the majority of health-care workers) | | | | 🡢 Template Letter to Advocate Hand Hygiene to Managers  🡢 Template Letter to communicate Hand Hygiene Initiatives to Managers  🡢 Guide to Implementation II.5 |
| 5.2a Chief executive officer | | No | 0 |  |
|  |  | Yes | 10 |  |
| 5.2b Medical director | | No | 0 |  |
|  |  | Yes | 5 |  |
| 5.2c Director of nursing | | No | 0 |  |
|  |  | Yes | 5 |  |
| **5.3**  Has a clear plan for the promotion of hand hygiene throughout the entire facility for the 5 May (Save Lives Clean Your Hands Annual Initiative) been established ? | | No | 0 | 🡢 Sustaining Improvement – Additional Activities for  Consideration by Health-Care Facilities  🡢 Guide to Implementation II.5 |
|  |  | Yes | 10 |  |
| **5.4**  Are systems for identification of Hand Hygiene Leaders from all disciplines in place? | | | |  |
| 5.4a A system for designation of Hand Hygiene champions11 | | No | 0 |  |
|  |  | Yes | 5 |  |
| 5.4b A system for recognition and utilisation of Hand Hygiene role models12 | | No | 0 |  |
|  |  | Yes | 5 |  |
| **5.5**  Regarding patient involvement in hand hygiene promotion: | | | | 🡢 Guidance on Engaging Patients and Patient Organizations in Hand Hygiene Initiatives  🡢 Guide to Implementation II.5 |
| 5.5a Are patients informed about the importance of hand hygiene? (e.g. with a leaflet) | | No | 0 |  |
|  |  | Yes | 5 |  |
| 5.5b Has a formalised programme of patient engagement been undertaken? | | No | 0 |  |
|  |  | Yes | 10 |  |
| **5.6**  Are initiatives to support local continuous improvement being applied in your facility, for example: | | | | 🡢 Sustaining Improvement – Additional Activities for  Consideration by Health-Care Facilities  🡢 Guide to Implementation II.5 |
| 5.6a Hand hygiene E-learning tools | | No | 0 |  |
|  |  | Yes | 5 |  |
| 5.6b A hand hygiene institutional target to be achieved is established each year | | No | 0 |  |
|  |  | Yes | 5 |  |
| 5.6c A system for intra-institutional sharing of reliable and tested local innovations | | No | 0 |  |
|  |  | Yes | 5 |  |
| 5.6d Communications that regularly mention hand hygiene e.g. facility newsletter, clinical meetings | | No | 0 |  |
|  |  | Yes | 5 |  |
| 5.6e System for personal accountability13 | | No | 0 |  |
|  |  | Yes | 5 |  |
| 5.6f A Buddy system14 for new employees | | No | 0 |  |
|  |  | Yes | 5 |  |
|  | **Institutional Safety Climate subtotal** | | **/100** |  |

**Infection Prevention and Control Assessment Framework**

World Health Organization. Infection prevention and control assessment framework at the facility level. <https://www.who.int/publications/i/item/WHO-HIS-SDS-2018.9>. Published 2018. Accessed July 26, 2024.

| **Core component 1: Infection Prevention and Control (IPC) programme** | | |
| --- | --- | --- |
| **Question** | **Answer** | **Score** |
| **1. Do you have an IPC programme?**3  Choose one answer | No | 0 |
|  | Yes, without clearly defined objectives | 5 |
|  | Yes, with clearly defined objectives and annual activity plan | 10 |
| **2. Is the IPC programme supported by an IPC team comprising of IPC professionals?**4  Choose one answer | No | 0 |
|  | Not a team, *only* an IPC focal person | 5 |
|  | Yes | 10 |
| **3. Does the IPC team have at least one full-time IPC professional or equivalent (nurse or doctor working 100% in IPC) available?** Choose one answer | No IPC professional available | 0 |
|  | No, *only* a part-time IPC professional available | 2.5 |
|  | Yes, one per > 250 beds | 5 |
|  | Yes, one per ≤ 250 beds | 10 |
| **4. Does the IPC team or focal person have dedicated time for IPC activities?** | No | 0 |
|  | Yes | 10 |
| **5. Does the IPC team include both doctors and nurses?** | No | 0 |
|  | Yes | 10 |
| **6. Do you have an IPC committee**5 **actively supporting the IPC team?** | No | 0 |
|  | Yes | 10 |

**7. Are any of the following professional groups represented/included in the IPC committee?**

| Senior facility leadership (for example, administrative director, chief executive officer [CEO], medical director) | No | 0 |
| --- | --- | --- |
|  | Yes | 5 |
| Senior clinical staff (for example, physician, nurse) | No | 0 |
|  | Yes | 2.5 |
| Facility management (for example, biosafety, waste, and those tasked with addressing water, sanitation, and hygiene [WASH]) | No | 0 |
|  | Yes | 2.5 |
| **8. Do you have clearly defined IPC objectives (that is, in specific critical areas)?**  Choose one answer | No | 0 |
|  | Yes, IPC objectives *only* | 2.5 |
|  | Yes, IPC objectives and measurable outcome indicators (that is, adequate measures for improvement) | 5 |
|  | Yes, IPC objectives, measurable outcome indicators and set future targets | 10 |

**9. Does the senior facility leadership show clear commitment and support for the IPC programme:**

| By an allocated budget specifically for the IPC programme (that is, covering IPC activities, including salaries)? | No | 0 |
| --- | --- | --- |
|  | Yes | 5 |
| By demonstrable support for IPC objectives and indicators within the facility (for example, at executive level meetings, executive rounds, participation in morbidity and mortality meetings)? | No | 0 |
|  | Yes | 5 |
| **10. Does your facility have microbiological laboratory support (either present on or off site) for routine day-to-day use?** Choose one answer | No | 0 |
|  | Yes, but not delivering results reliably (timely and of sufficient quality) | 5 |
|  | Yes, and delivering results reliably (timely and of sufficient quality) | 10 |
| **Subtotal score /100** | | |

| **Core component 2: Infection Prevention and Control (IPC) guidelines** | | |
| --- | --- | --- |
| **Question** | **Answer** | **Score** |
| **1. Does your facility have the expertise (in IPC and/or infectious diseases) for developing or adapting guidelines?** | No | 0 |
|  | Yes | 7.5 |
| **2. Does your facility have guidelines available for:** | | |
| Standard precautions? | No | 0 |
|  | Yes | 2.5 |
| Hand hygiene? | No | 0 |
|  | Yes | 2.5 |
| Transmission-based precautions?6 | No | 0 |
|  | Yes | 2.5 |
| Outbreak management and preparedness? | No | 0 |
|  | Yes | 2.5 |
| Prevention of surgical site infection?7 | No | 0 |
|  | Yes | 2.5 |
| Prevention of vascular catheter-associated bloodstream infections? | No | 0 |
|  | Yes | 2.5 |
| Prevention of hospital-acquired pneumonia ([HAP]; all types of HAP, including (but not exclusively) ventilator-associated pneumonia)? | No | 0 |
|  | Yes | 2.5 |
| Prevention of catheter-associated urinary tract infections? | No | 0 |
|  | Yes | 2.5 |
| Prevention of transmission of multidrug-resistant (MDR) pathogens? | No | 0 |
|  | Yes | 2.5 |
| Disinfection and sterilization? | No | 0 |
|  | Yes | 2.5 |
| Health care worker protection and safety8 | No | 0 |
|  | Yes | 2.5 |
| Injection safety? | No | 0 |
|  | Yes | 2.5 |
| Waste management? | No | 0 |
|  | Yes | 2.5 |
| Antibiotic stewardship?9 | No | 0 |
|  | Yes | 2.5 |

| **3. Are the guidelines in your facility consistent with national/international guidelines (if they exist)?** | No | 0 |
| --- | --- | --- |
|  | Yes | 10 |
| **4. Is implementation of the guidelines adapted**10 **according to the local needs and resources while maintaining key IPC standards?** | No | 0 |
|  | Yes | 10 |
| **5. Are frontline health care workers involved in both planning and executing the implementation of IPC guidelines in addition to IPC personnel?** | No | 0 |
|  | Yes | 10 |
| **6. Are relevant stakeholders (for example, lead doctors and nurses, hospital managers, quality management) involved in the development and adaptation of the IPC guidelines in addition to IPC personnel?** | No | 0 |
|  | Yes | 7.5 |
| **7. Do health care workers receive specific training related to new or updated IPC guidelines introduced in the facility?** | No | 0 |
|  | Yes | 10 |
| **8. Do you regularly monitor the implementation of at least some of the IPC guidelines in your facility?** | No | 0 |
|  | Yes | 10 |
| **Subtotal score /100** | | |

| **Core component 3: Infection Prevention and Control (IPC) education and training** | | |
| --- | --- | --- |
| **Question** | **Answer** | **Score** |
| **1. Are there personnel with the IPC expertise (in IPC and/or infectious diseases) to lead IPC training?** | No | 0 |
|  | Yes | 10 |
| **2. Are there additional non-IPC personnel with adequate skills to serve as trainers and mentors (for example, link nurses or doctors, champions)?**  Choose one answer | No | 0 |
|  | Yes | 10 |
| **3. How frequently do health care workers receive training regarding IPC in your facility?**  Choose one answer | Never or rarely | 0 |
|  | New employee orientation *only* for health care workers | 5 |
|  | New employee orientation and regular (at least annually) IPC training for health care workers offered but not mandatory | 10 |
|  | New employee orientation and regular (at least annually) mandatory IPC training for all health care workers | 15 |
| **4. How frequently do cleaners and other personnel directly involved in patient care receive training regarding IPC in your facility?** Choose one answer | Never or rarely | 0 |
|  | New employee orientation *only* for other personnel | 5 |
|  | New employee orientation and regular (at least annually) training for other personnel offered but not mandatory | 10 |
|  | New employee orientation and regular (at least annually) mandatory IPC training for other personnel | 15 |
| **5. Does administrative and managerial staff receive general training regarding IPC in your facility?**  Choose one answer | No | 0 |
|  | Yes | 5 |
| **6. How are health care workers and other personnel trained?**  Choose one answer | No training available | 0 |
|  | Using written information and/or oral instruction and/or e-learning *only* | 5 |
|  | Includes *additional* interactive training sessions (for example, simulation and/or bedside training) | 10 |
| **7. Are there periodic evaluations of the effectiveness of training programmes (for example, hand hygiene audits, other checks on knowledge)?**  Choose one answer | No | 0 |
|  | Yes, but not regularly | 5 |
|  | Yes, regularly (at least annually) | 10 |
| **8. Is IPC training integrated in the clinical practice and training of other specialties (for example, training of surgeons involves aspects of IPC)?**  Choose one answer | No | 0 |
|  | Yes, in some disciplines | 5 |
|  | Yes, in all disciplines | 10 |
| **9. Is there specific IPC training for patients or family members to minimize the potential for health care-associated infections**  **(for example, immunosuppressed patients, patients with invasive devices, patients with multidrug-resistant infections)?** | No | 0 |
|  | Yes | 5 |
| **10. Is ongoing development/education offered for IPC staff (for example, by regularly attending conferences, courses)?** | No | 0 |
|  | Yes | 10 |
| **Subtotal score /100** | | |

| **Core component 4: Health care-associated infection (HAI) surveillance** | | |
| --- | --- | --- |
| **Question** | **Answer** | **Score** |

**Organization of surveillance**

| **1. Is surveillance a defined component of your IPC programme?** | No | 0 |
| --- | --- | --- |
|  | Yes | 5 |
| **2. Do you have personnel responsible for surveillance activities?** | No | 0 |
|  | Yes | 5 |
| **3. Have the professionals responsible for surveillance activities been trained in basic epidemiology, surveillance and IPC (that is, capacity to oversee surveillance methods, data management and interpretation)?** | No | 0 |
|  | Yes | 5 |
| **4. Do you have informatics/IT support to conduct your surveillance (for example, equipment, mobile technologies, electronic health records)?** | No | 0 |
|  | Yes | 5 |

**Priorities for surveillance - defined according to the scope of care**

| **5. Do you go through a prioritization exercise to determine the HAIs to be targeted for surveillance according to the local context (that is, identifying infections that are major causes of morbidity and mortality in the facility)?**11 | No | 0 |
| --- | --- | --- |
|  | Yes | 5 |

**6. In your facility is surveillance conducted for:**

| Surgical site infections?12 | No | 0 |
| --- | --- | --- |
|  | Yes | 2.5 |
| Device-associated infections (for example, catheter-associated urinary tract infections, central line-associated bloodstream infections, peripheral-line associated bloodstream infections, ventilator-associated pneumonia)? | No | 0 |
|  | Yes | 2.5 |
| Clinically-defined infections (for example, definitions based only on clinical signs or symptoms in the absence of microbiological testing)? | No | 0 |
|  | Yes | 2.5 |
| Colonization or infections caused by multidrug-resistant13 pathogens according to your local epidemiological situation? | No | 0 |
|  | Yes | 2.5 |
| Local priority epidemic-prone infections (for example, norovirus, influenza, tuberculosis [TB], severe acute respiratory syndrome [SARS], Ebola, Lassa fever)? | No | 0 |
|  | Yes | 2.5 |
| Infections in vulnerable populations (for example, neonates, intensive care unit, immunocompromised, burn patients)?14 | No | 0 |
|  | Yes | 2.5 |
| Infections that may affect health care workers in clinical, laboratory, or other settings (for example, hepatitis B or C, human immunodeficiency virus [HIV], influenza)? | No | 0 |
|  | Yes | 2.5 |
| **7. Do you regularly evaluate if your surveillance is in line with the current needs and priorities of your facility?**11 | No | 0 |
|  | Yes | 5 |

**Methods of surveillance**

| **8. Do you use reliable surveillance case definitions (defined numerator and denominator according to international definitions [e.g. CDC NHSN/ECDC]**15 **or if adapted, through an evidence-based adaptation process and expert consultation?** | No | 0 |
| --- | --- | --- |
|  | Yes | 5 |
| **9. Do you use standardized data collection methods (for example, active prospective surveillance) according to international surveillance protocols (for example, CDC NHSN/ECDC) or if adapted, through an evidence-based adaptation process and expert consultation?** | No | 0 |
|  | Yes | 5 |
| **10. Do you have processes in place to regularly review data quality (for example, assessment of case report forms, review**  **of microbiology results, denominator determination, etc.)?** | No | 0 |
|  | Yes | 5 |
| **11. Do you have adequate microbiology and laboratory capacity to support surveillance?**  Choose one answer | No | 0 |
|  | Yes, can differentiate gram-positive/negative strains but cannot identify pathogens | 2.5 |
|  | Yes, can reliably identify pathogens (for example, isolate identification) in a timely manner | 5 |
|  | Yes, can reliably identify pathogens and antimicrobial drug resistance patterns (that is, susceptibilities) in a timely manner | 10 |

**Information analysis and dissemination/data use, linkage, and governance**

| **12. Are surveillance data used to make tailored unit/facility-based plans for the improvement of IPC practices?** | No | 0 |
| --- | --- | --- |
|  | Yes | 5 |
| **13. Do you analyze antimicrobial drug resistance on a regular basis (for example, quarterly/half-yearly/annually)?** | No | 0 |
|  | Yes | 5 |
| **14. Do you regularly (for example, quarterly/half-yearly/annually) feedback up-to-date surveillance information to:** | | |
| Frontline health care workers (doctors/nurses)? | No | 0 |
|  | Yes | 2.5 |
| Clinical leaders/heads of department | No | 0 |
|  | Yes | 2.5 |
| IPC committee | No | 0 |
|  | Yes | 2.5 |
| Non-clinical management/administration (chief executive officer/chief financial officer)? | No | 0 |
|  | Yes | 2.5 |
| **15. How do you feedback up-to-date surveillance information? (at least annually)**  Choose one answer | No feedback | 0 |
|  | By written/oral information *only* | 2.5 |
|  | By presentation and interactive problem-orientated solution finding | 7.5 |
| **Subtotal score /100** | | |

| **Core component 5: Multimodal strategies16 for implementation of infection prevention and control (IPC) interventions** | | |
| --- | --- | --- |
| **Question** | **Answer** | **Score** |
| **1. Do you use multimodal strategies16 to implement IPC interventions?** | No | 0 |
|  | Yes | 15 |
| **2. Do your multimodal strategies include any or all of the following elements:**  Choose one answer (the most accurate) per element | **System change** | 0 |
|  | Element not included in multimodal strategies | 0 |
|  | Interventions to ensure the necessary infrastructure and continuous availability of supplies are in place | 5 |
|  | Interventions to ensure the necessary infrastructure and continuous availability of supplies are in place and addressing ergonomics17 and accessibility, such as the best placement of central venous catheter set and tray | 10 |
|  | **Education and training** |  |
|  | Element not included in multimodal strategies | 0 |
|  | Written information and/or oral instruction and/or e-learning *only* | 5 |
|  | *Additional* interactive training sessions (includes simulation and/or bedside training) | 10 |
|  | **Monitoring and feedback** |  |
|  | Element not included in multimodal strategies | 0 |
|  | Monitoring compliance with process or outcome indicators (for example, audits of hand hygiene or catheter practices) | 5 |
|  | Monitoring compliance and providing timely feedback of monitoring results to health care workers and key players | 10 |
|  | **Communications and reminders** |  |
|  | Element not included in multimodal strategies | 0 |
|  | Reminders, posters, or other advocacy/awareness-raising tools to promote the intervention | 5 |
|  | *Additional* methods/initiatives to improve team communication across units and disciplines (for example, by establishing regular case conferences and feedback rounds) | 10 |
|  | **Safety climate and culture change** |  |
|  | Element not included in multimodal strategies | 0 |
|  | Managers/leaders show visible support and act as champions and role models, promoting an adaptive approach18 and strengthening a culture that supports IPC, patient safety and quality | 5 |
|  | *Additionally*, teams and individuals are empowered so that they perceive ownership of the intervention (for example, by participatory feedback rounds) | 10 |

| **3. Is a multidisciplinary team used to implement IPC multimodal strategies?** | No | 0 |
| --- | --- | --- |
|  | Yes | 15 |
| **4. Do you regularly link to colleagues from quality improvement and patient safety to develop and promote IPC multimodal strategies?** | No | 0 |
|  | Yes | 10 |
| **5. Do these strategies include bundles**19 **or checklists?** | No | 0 |
|  | Yes | 10 |
| **Subtotal score /100** | | |

| **Core component 6: Monitoring/audit of IPC practices and feedback** | | |
| --- | --- | --- |
| **Question** | **Answer** | **Score** |
| **1. Do you have trained personnel responsible for monitoring/audit of IPC practices and feedback?** | No | 0 |
|  | Yes | 10 |
| **2. Do you have a well-defined monitoring plan with clear goals, targets and activities (including tools to collect data in a systematic way)?** | No | 0 |
|  | Yes | 7.5 |
| **3. Which processes and indicators do you monitor in your facility?**  Tick all that apply | None | 0 |
|  | Hand hygiene compliance (using the WHO hand hygiene observation tool20 or equivalent) | 5 |
|  | Intravascular catheter insertion and/or care | 5 |
|  | Wound dressing change | 5 |
|  | Transmission-based precautions and isolation to prevent the spread of multidrug resistant organisms (MDRO) | 5 |
|  | Cleaning of the ward environment | 5 |
|  | Disinfection and sterilization of medical equipment/instruments | 5 |
|  | Consumption/usage of alcohol-based handrub or soap | 5 |
|  | Consumption/usage of antimicrobial agents | 5 |
|  | Waste management | 5 |
| **4. How frequently is the *WHO Hand Hygiene Self-Assessment Framework Survey*** 21 **undertaken?**  Choose one answer | Never | 0 |
|  | Periodically, but no regular schedule | 2.5 |
|  | At least annually | 5 |
| **5. Do you feedback auditing reports (for example, feedback on hand hygiene compliance data or other processes) on the state of the IPC activities/performance?**  Tick all that apply | No reporting | 0 |
|  | Yes, within the IPC team | 2.5 |
|  | Yes, to department leaders and managers in the areas being audited | 2.5 |
|  | Yes, to frontline health care workers | 2.5 |
|  | Yes, to the IPC committee or quality of care committees or equivalent | 2.5 |
|  | Yes, to hospital management and senior administration | 2.5 |
| **6. Is the reporting of monitoring data undertaken regularly (at least annually)?** | No | 0 |
|  | Yes | 10 |
| **7. Are monitoring and feedback of IPC processes and indicators performed in a “blame-free” institutional culture aimed at improvement and behavioural change?** | No | 0 |
|  | Yes | 5 |
| **8. Do you assess safety cultural factors in your facility (for example, by using other surveys such as HSOPSC, SAQ, PSCHO, HSC**22**)** | No | 0 |
|  | Yes | 5 |
| **Subtotal score /100** | | |

| **Core component 7: Workload, staffing and bed occupancy**23 | | |
| --- | --- | --- |
| **Question** | **Answer** | **Score** |

**Staffing**

| **1. Are appropriate staffing levels assessed in your facility according to patient workload using national standards or a standard staffing needs assessment tool such as the *WHO Workload indicators of staffing need***24 **method?** | No | 0 |
| --- | --- | --- |
|  | Yes | 5 |
| **2. Is an agreed (that is, WHO or national) ratio of health care workers to patients**25 **maintained across your facility?**  Choose one answer | No | 0 |
|  | Yes, for staff in less than 50% of units | 5 |
|  | Yes, for staff in more than 50% of units | 10 |
|  | Yes, for all health care workers in the facility | 15 |
| **3. Is a system in place in your facility to act on the results of the staffing needs assessments when staffing levels are deemed to be too low?** | No | 0 |
|  | Yes | 10 |

**Bed occupancy**

| **4. Is the design of wards in your facility in accordance with international standards**26 **regarding bed capacity?**  Choose one answer | No | 0 |
| --- | --- | --- |
|  | Yes, but *only* in certain departments | 5 |
|  | Yes, for all departments (including emergency department and pediatrics) | 15 |
| **5. Is bed occupancy in your facility kept to one patient per bed?**  Choose one answer | No | 0 |
|  | Yes, but *only* in certain departments | 5 |
|  | Yes, for all units (including emergency departments and pediatrics) | 15 |
| **6. Are patients in your facility placed in beds standing in the corridor outside of the room (including beds in the emergency department)?** Choose one answer | Yes, more frequently than twice a week | 0 |
|  | Yes, less frequently than twice a week | 5 |
|  | No | 15 |
| **7. Is adequate spacing of > 1 meter between patient beds ensured in your facility?**  Choose one answer | No | 0 |
|  | Yes, but *only* in certain departments | 5 |
|  | Yes, for all departments (including emergency department and pediatrics) | 15 |
| **8. Is a system in place in your facility to assess and respond when adequate bed capacity is exceeded?**  Choose one answer | No | 0 |
|  | Yes, this is the responsibility of the head of department | 5 |
|  | Yes, this is the responsibility of the hospital administration/ management | 10 |
| **Subtotal score /100** | | |

| **Core component 8: Built environment, materials and equipment for IPC at the facility level**27 | | |
| --- | --- | --- |
| **Question** | **Answer** | **Score** |

**Water**

| **1. Are water services available at all times and of sufficient quantity for all uses (for example, hand washing, drinking, personal hygiene, medical activities, sterilization, decontamination, cleaning and laundry)?**  Choose one answer | No, available on average < 5 days per week | 0 |
| --- | --- | --- |
|  | Yes, available on average ≥ 5 days per week or every day but not of sufficient quantity | 2.5 |
|  | Yes, every day and of sufficient quantity | 7.5 |
| **2. Is a reliable safe drinking water station present and accessible for staff, patients and families at all times and in all locations/wards?** Choose one answer | No, not available | 0 |
|  | Sometimes, or only in some places or not available for all users | 2.5 |
|  | Yes, accessible at all times and for all wards/groups | 7.5 |

**Hand hygiene and sanitation facilities**

| **3. Are functioning hand hygiene stations (that is, alcohol-based handrub solution or soap and water and clean single-use towels) available at all points of care?**  Choose one answer | No, not present | 0 |
| --- | --- | --- |
|  | Yes, stations present, but supplies are not reliably available | 2.5 |
|  | Yes, with reliably available supplies | 7.5 |
| **4. In your facility, are ≥ 4 toilets or improved latrines**28 **available for outpatient settings or ≥ 1 per 20 users for inpatient settings?** Choose one answer | Less than required number of toilets or latrines available and functioning | 0 |
|  | Sufficient number present but not all functioning | 2.5 |
|  | Sufficient number present and functioning | 7.5 |

**Power supply, ventilation and cleaning**

| **5. In your health care facility, is sufficient energy/power supply available at day and night for all uses (for example, pumping and boiling water, sterilization and decontamination, incineration or alternative treatment technologies, electronic medical devices, general lighting of areas where health care procedures are performed to ensure safe provision of health care and lighting of toilet facilities and showers)?** Choose one answer | No | 0 |
| --- | --- | --- |
|  | Yes, sometimes or only in some of the mentioned areas | 2.5 |
|  | Yes, always and in all mentioned areas | 5 |
| **6. Is functioning environmental ventilation (natural or mechanical**29**) available in patient care areas?** | No | 0 |
|  | Yes | 5 |
| **7. For floors and horizontal work surfaces, is there an accessible record of cleaning, signed by the cleaners each day?**  Choose one answer | No record of floors and surfaces being cleaned | 0 |
|  | Record exists, but is not completed and signed daily or is outdated | 2.5 |
|  | Yes, record completed and signed daily | 5 |
| **8. Are appropriate and well-maintained materials for cleaning (for example, detergent, mops, buckets, etc.) available?** Choose one answer | No materials available | 0 |
|  | Yes, available but not well maintained | 2.5 |
|  | Yes, available and well-maintained | 5 |

**Patient placement and personal protective equipment (PPE) in health care settings**

| **9. Do you have single patient rooms or rooms for cohorting**30 **patients with similar pathogens if the number of isolation rooms is insufficient (for example, TB, measles, cholera, Ebola, SARS)?**31 Choose one answer | No | 0 |
| --- | --- | --- |
|  | No single rooms but rather rooms suitable for patient cohorting available | 2.5 |
|  | Yes, single rooms are available | 7.5 |
| **10. Is PPE**32 **available at all times and in sufficient quantity for all uses for all health care workers?**  Choose one answer | No | 0 |
|  | Yes, but not continuously available in sufficient quantities | 2.5 |
|  | Yes, continuously available in sufficient quantities | 7.5 |

**Medical waste management and sewage**

| **11. Do you have functional waste collection containers for**  **non-infectious (general) waste, infectious waste and, sharps waste in close proximity to all waste generation points?**  Choose one answer | No bins or separate sharps disposal | 0 |
| --- | --- | --- |
|  | Separate bins present but lids missing or more than 3/4 full; only two bins (instead of three); or bins at some but not all waste generation points | 2.5 |
|  | Yes | 5 |
| **12. Is a functional burial pit/fenced waste dump or municipal pick-up available for disposal of non-infectious (non-hazardous/ general waste)?**  Choose one answer | No pit or other disposal method used | 0 |
|  | Pit in facility but insufficient dimensions; pits/dumps overfilled or not fenced/locked; or irregular municipal waste pick up | 2.5 |
|  | Yes | 5 |
| **13. Is an incinerator or alternative treatment technology for the treatment of infectious and sharp waste (for example, an autoclave) present (either present on or off site and operated by a licensed waste management service), functional and of a sufficient capacity?** Choose one answer | No, none present | 0 |
|  | Present, but not functional | 1 |
|  | Yes | 5 |
| **14. Is a wastewater treatment system (for example, septic tank followed by drainage pit) present (either on or off site) and functioning reliably?**  Choose one answer | No, not present | 0 |
|  | Yes, but not functioning reliably | 2.5 |
|  | Yes and functioning reliably | 5 |

**Decontamination and sterilization**

| **15. Does your health care facility provide a dedicated decontamination area and/or sterile supply department (either present on or off site and operated by a licensed decontamination management service) for the decontamination and sterilization**  **of medical devices and other items/equipment?**  Choose one answer | No, not present | 0 |
| --- | --- | --- |
|  | Yes, but not functioning reliably | 2.5 |
|  | Yes and functioning reliably | 5 |
| **16. Do you reliably have sterile and disinfected equipment ready for use?**  Choose one answer | No, available on average < five days per week | 0 |
|  | Yes, available on average ≥ five days per week or every day, but not of sufficient quantity | 2.5 |
|  | Yes, available every day and of sufficient quantity | 5 |
| **17. Are disposable items available when necessary? (for example, injection safety devices, examination gloves)**  Choose one answer | No, not available | 0 |
|  | Yes, but *only* sometimes available | 2.5 |
|  | Yes, continuously available | 5 |
| **Subtotal score /100** | | |

**Interpretation: A three-step process . 1. Add up your points**

|  | **Score** |
| --- | --- |
| **Section (Core component)** | **Subtotals** |
| 1. IPC programme |  |
| 2. IPC guidelines |  |
| 3. IPC education and training |  |
| 4. HAI surveillance |  |
| 5. Multimodal strategies |  |
| 6. Monitoring/audits of IPC practices and feedback |  |
| 7. Workload, staffing and bed occupancy |  |
| 8. Built environment, materials and equipment for IPC at the facility level |  |
| **Final total score /800** | |

**2. Determine the assigned “IPC level” in your facility using the total score from Step 1**

| **Total score** (range) | **IPC level** |
| --- | --- |
| 0–200 | Inadequate |
| 201–400 | Basic |
| 401–600 | Intermediate |
| 601–800 | Advanced |

**3. Review the framework results and develop an action plan**

Review the areas identified by this evaluation as requiring improvement in your facility and develop an action plan to address them. To undertake this task, consult the WHO Interim practical manual supporting implementation of the WHO Guidelines on Core Components of Infection Prevention and Control Programmes2 which will provide you with guidance, templates, tips, and examples from around the world as well as with a list of relevant IPC improvement tools. Keep a copy of this assessment to compare with repeated uses in the future.
